# Genotyping of Soybean Cultivars With Medium-Density Array Reveals the Population Structure and QTNs Underlying Maturity and Seed Traits

**DOI:** 10.3389/fpls.2018.00610

**Published:** 2018-05-09

**Authors:** Ya-ying Wang, Yu-qiu Li, Hong-yan Wu, Bo Hu, Jia-jia Zheng, Hong Zhai, Shi-xiang Lv, Xin-lei Liu, Xin Chen, Hong-mei Qiu, Jiayin Yang, Chun-mei Zong, De-zhi Han, Zi-xiang Wen, De-chun Wang, Zheng-jun Xia

**Affiliations:** ^1^Key Laboratory of Soybean Molecular Design Breeding, Northeast Institute of Geography and Agroecology, Chinese Academy of Sciences, Harbin, China; ^2^University of Chinese Academy of Sciences, Beijing, China; ^3^Soybean Research Institute, Jilin Academy of Agricultural Sciences, Changchun, China; ^4^Heilongjiang Academy of Agricultural Sciences, Harbin, China; ^5^Jiangsu Academy of Agricultural Sciences, Nanjing, China; ^6^Huaiyin Institute of Agricultural Sciences in Xuhuai Region of Jiangsu Province, Huaian, China; ^7^Mudanjiang Branch of Heilongjiang Academy of Agricultural Sciences, Mudanjiang, China; ^8^Heihe Branch of Heilongjiang Academy of Agricultural Sciences, Heihe, China; ^9^Department of Plant, Soil and Microbial Sciences, Michigan State University, East Lansing, MI, United States

**Keywords:** soybean, GWAS, flowering time, protein content, oil content, population structure, FarmCPU

## Abstract

Soybean was domesticated about 5,000 to 6,000 years ago in China. Although genotyping technologies such as genotyping by sequencing (GBS) and high-density array are available, it is convenient and economical to genotype cultivars or populations using medium-density SNP array in genetic study as well as in molecular breeding. In this study, 235 cultivars, collected from China, Japan, USA, Canada and some other countries, were genotyped using SoySNP8k iSelect BeadChip with 7,189 single nucleotide polymorphisms (SNPs). In total, 4,471 polymorphic SNP markers were used to analyze population structure and perform genome-wide association study (GWAS). The most likely K value was 7, indicating this population can be divided into 7 subpopulations, which is well in accordance with the geographic origins of cultivars or accession studied. The LD decay rate was estimated at 184 kb, where r^2^ dropped to half of its maximum value (0.205). GWAS using FarmCPU detected a stable quantitative trait nucleotide (QTN) for hilum color and seed color, which is consistent with the known loci or genes. Although no universal QTNs for flowering time and maturity were identified across all environments, a total of 30 consistent QTNs were detected for flowering time (R1) or maturity (R7 and R8) on 16 chromosomes, most of them were corresponding to known *E1* to *E4* genes or QTL region reported in SoyBase (soybase.org). Of 16 consistent QTNs for protein and oil contents, 11 QTNs were detected having antagonistic effects on protein and oil content, while 4 QTNs soly for oil content, and one QTN soly for protein content. The information gained in this study demonstrated that the usefulness of the medium-density SNP array in genotyping for genetic study and molecular breeding.

## Introduction

Soybean [*Glycine max* (L.) Merr.] is one of important crops worldwide, providing a sustainable source of high-quality protein feed and vegetable oil. Soybean was domesticated in China more than 5,000–6,000 years ago. Soybean can grow across a wide range of latitudes from 50°N to 35°S (Norman, 1978). Soybean yield related traits such as flowering, maturity and protein/oil contents are quantitatively inherited traits controlled by internal and external factors (Xia et al., 2013).

Each soybean cultivar adapts to a limited latitudinal region for its maximal yield since soybean is a short day plants with photoperiod sensitivity (Xia et al., 2012b). Flowering time and maturity are important agronomic traits related to soybean adaptability and productivity. More than 200 loci or genes have been mapped to control flowering time in soybean (SoyBase, www.soybase.org). Previous studies identified eleven major-effect loci affecting flowering and maturity in soybean, which have been designated as *E1* to *E10*, and the *J* locus for “long juvenile period” (Bernard, 1971; Buzzell, 1971; Buzzell and Voldeng, 1980; McBlain and Bernard, 1987; Ray et al., 1995; Bonato and Vello, 1999; Cober and Voldeng, 2001; Cober et al., 2010; Kong et al., 2014; Samanfar et al., 2017). Of these genes, *E1, E2, E3, E4, E6, E9, E10*, and *J* have been cloned and functionally characterized (Liu et al., 2008; Watanabe et al., 2009, 2011; Xia et al., 2012a; Zhai et al., 2014a; Zhao et al., 2016; Lu et al., 2017; Samanfar et al., 2017). *E1* encodes a nuclear-localized B3 domain-containing protein, suppresses both *GmFT2a* and *GmFT5a* expression, two *FT* orthologs promoting early flowering in soybean (Xia et al., 2012a). *E1* expression is suppressed in short day, which is regarded as the main factor for soybean being a short day plant (Xia et al., 2012a; Zhai et al., 2015; Zhang et al., 2016). *E2* encodes a homolog of *GIGANTEA*, controls soybean flowering through regulation of *GmFT2a* expression but not *GmFT5a* (Watanabe et al., 2011). *E3* and *E4* are *Phytochrome A* (*PHYA*) genes of *GmPHYA3* and *GmPHYA2* (Liu et al., 2008; Watanabe et al., 2009). Various allelic combinations of *E1, E3* or *E4* lead to various photoperiod insensitivity, enabling soybean to adapt to high-latitude environments (Zhai et al., 2014b). *J* loci is identified as the ortholog of *Arabidopsis thaliana EARLY FLOWERING 3* (*ELF3*), which control flowering time through regulation of *E1* expression (Lu et al., 2017). Higher *E1* expression in short day enables soybean to grow in the area of lower latitude near equator. *E9* and *E10* are *GmFT2a* and *GmFT4, FT* homolog of Arabidopsis (Zhai et al., 2014a; Zhao et al., 2016). Apart from negative report on existence of *E5* loci (Dissanayaka et al., 2016), molecular identities of *E7* and *E8* are still unknown. Many quantitative trait loci (QTL) or quantitative trait nucleotide (QTN) related to soybean flowering time (first flowering, R1) and maturity have also been documented at SoyBase (http://soybase.org). Many genes or QTL might regulate flowering time through regulation of the expression of the *E1* gene (Zhai et al., 2015).

Soybean seed compositions traits such as protein and oil contents are important quality traits in breeding programs. Patil et al. (2017) reviewed molecular mapping and genomic of soybean seed protein, and concluded genetic improvement of soybean protein meal is a complex process because of negative correlation with oil, yield, and the temperature (Patil et al., 2017). Major QTL were repeated detected on chromosome (20 (LG I) and 15 (LG E) (Patil et al., 2017). Leamy et al. (2017) studied seed composition traits in wild soybean (*Glycine soja*) and found 29 SNPs located on ten different chromosomes that are significantly associated with the seven seed composition traits, of which eight SNPs co-localized with QTLs previously uncovered in linkage or association mapping studies conducted with cultivated soybean samples (Leamy et al., 2017). Zhou et al. (2015) mapped major QTN for protein on chromosome 13, 3, 17, 12, 11, and 15 using a 302 accessions (Zhou et al., 2015). More than 100 quantitative trait loci (QTLs) for soybean oil content have been documented at SoyBase (https://www.soybase.org). Cao et al. (2017) found 8 QTLs explained a range of phenotypic variance from 6.3 to 26.3% using RIL population, and *qOil-5-1, qOil-10-1*, and *qOil-14-1* were detected in different environments (Cao et al., 2017). And *qOil-5-1* was also detected using natural population and further localized to a linkage disequilibrium block region of approximately 440 kb (Zhang et al., 2017). *WRINKLED1*(*WRI1*), *LEAFY COTYLEDON1* (*LEC1*), and *LEC2* are involved in the regulatory pathways modulating seed oil content in Arabidopsis. However, their homologs have been modified in the palaeopolyploid soybean, each exhibiting similar intensities of purifying selection to their respective duplicates since these pairs were formed by a 13 mya (million years ago) whole-genome duplication (WGD) event (Zhang et al., 2017).

Recently, researchers have been applied GWAS in soybean (Bandillo et al., 2015; Wen et al., 2015; Zhang et al., 2015, 2016; Zhou et al., 2015; Contreras-Soto et al., 2017; Fang et al., 2017). Zhang et al. (2015) revealed that genetic loci underlying some agronomically important traits, such as days to flowering, days to maturity, duration of flowering-to-maturity, and plant height in early maturity soybean (Zhang et al., 2015). The ability of GWAS to capture one trait often depends on the frequency of the accessions with contrast phenotypic value in the population being investigated. Recently, as the great advance in sequencing technology, genotyping by sequencing (GBS) has been a choice over other genotyping method, SNP array and traditional SSR markers.

In comparison of traditional linage analysis, genome-wide association study (GWAS) takes advantage of more historic recombination events that have occurred within natural populations. GWAS has been widely applied to crop plants such as maize (Tian et al., 2011), rice (Huang et al., 2010; Ma et al., 2016). However, in rice, recently studies demonstrates the power of GWAS in combination of biparental association mapping and fine-mapping in dissect agronomic important trait (Huang et al., 2010; Ma et al., 2016).

In this study, we genotyped 235 cultivars using Illumina SoySNP8k iSelect BeadChip; and 4471 core SNP markers were selected. A relatively complex population structure (K = 7) was revealed. GWAS were performed to identify the QTN associated with flowering time and the protein/oil contents using FarmCPU. More than 30 QTN were identified under multiple environments for flowering time and maturity; while 16 consistent QTNs were detected for protein and oil contents.

## Materials and methods

### Cultivars and growth condition

A set of 235 cultivars collected from China, Japan, USA, and Canada were mainly obtained from the Gene Resource Center of Jilin Academy of Agricultural Sciences, China. The origin and other traits for these cultivars are listed in Table S1.

### Phenotypic observation

Soybean accessions were evaluated for photoperiodic responses at six geographic locations: (1), Harbin (hereafter termed as HRB): Research field at the Campus of Northeast Institute of Geography and Agroecology, Harbin, Heilongjiang (45°70′N, 126°64′E); (2), Mudanjiang (hereafter termed as MDJ): Mudanjiang Research Station, Heilongjiang Academy of Agricultural Science (44°42′N, 129°52′E); (3) Gongzhuling (hereafter termed as GZL): Gongzhuling Research Station, Jilin Academy of Agricultural Science, Gongzhuling, Jilin (43°53′N, 124°84′E); (4) Jinan (JN): Campus of Shandong Normal University, Jinan, Shandong (36°66′N,117° 17′E); (5) Huaian (hereafter termed as HA): Huaiyin Research Station, Jiangsu Academy of Agricultural Science, Huaian, Jiangsu (33°57′N, 119°04′E); (6) Nanjing (hereafter termed as NJ): Luhe Research Station, Jiangsu Academy of Agricultural Science, Nanjing, Jiangsu (32°31′N, 118°82′E). At least 15 plants for each cultivar or accession per geographic location were grown in a single row with 20 cm apart for phenotypic evaluation. Days from planting to flowering (R1) and maturity (R7 and R8) were recorded according to Fehr's description (Fehr et al., 1971). R1 refers the beginning of bloom (the opening of the first flower at any node on the main stem). R7 represents the beginning of maturity (one normal pod on the main stem has reached its mature pod color, normally brown or tan); R8 stands for full maturity (95 percent of the pods having reached their mature pod color). For a given cultivar, each specific R stage is defined only when at least 50% of individual plants reached that stage.

Seed were harvested upon maturity. In HRB, GZL, MDJ locations, cultivars that did not reach mature stage (R8) were precluded for maturity and protein/oil content.

Seed coat or hilum color were classified into four groups and coded as follows: (1) yellow or yellowish; (2) green or light brown; (3) brown; (4) black. Seed-weight (100-seedweight) was determined by weighing 3 different set of randomly selected 100 seeds for each cultivar or accession. Seed protein and oil contents of cultivars were measured using MATRIX-I FT-NIR spectrometer (Bruker). The protein or oil contents were measured three times using different bulk seeds of a given cultivar.

The heritability estimates were calculated using variance components obtained by lme4 of R package (Fehr, 1987).

### Genotyping with SNP markers

DNA was extracted from fresh leaves using the hexadecyltrimethylammonium bromide (CTAB) method with slight modification (Murray and Thompson, 1980; Xia et al., 2007). Due to availability of financial budget, cultivars were divided into two batches (95 cultivars and 140 cultivars) to proceed genotyping. Genotyping using Illumina SoySNP8k iSelect BeadChip (Akond et al., 2013; Yang et al., 2017), which contained a total of 7,189 SNPs and was specifically manufactured by Infinium HD Ultra. SNP genotyping was performed with the Illumina Iscan platform (Illumina, Inc., San Diego, CA). A series of procedures, such as incubation, DNA amplification, preparation of bead assay, hybridization of samples for the bead assay, extension, staining of samples, and imaging of the bead assay, were conducted following previously reported methods (Song et al., 2013). The SNP alleles were called with the Genome Studio Genotyping module (Illumina, Inc.) (Song et al., 2013), and SNP data is available at ftp://159.226.208.134/public/SNP_data.zip (Data Sheet 1).

### Population structure analysis and GWAS

Population structure analysis was performed using STRUCTURE (Pritchard et al., 2000) and to choose the appropriate number of inferred clusters to model the data, 5 independent runs were performed for each K cluster (2 < K < 13, the length of the burn-in is 10,000, the length of MCMC(Markov chain Monte Carlo) is 10,000). After several attempts, we found that our parameter set was sufficient, longer length of burn-in and MCMC did not change the result significantly. Furthermore, population structure was assessed for K values ranging from 2 to 13 on the entire panel using high quality SNPs. The calculation method of STRUCTURE is based on the Bayesian model. For the simulation result of each K value, STRUCTURE will correspondingly produce the log maximum likelihood value, “LnP(D).” As LnP(D) increases, the K value is closer to the real case. The simulation result with largest LnP(D) and smallest K value is the optimal result (Evanno et al., 2005). The neighbor-joining tree was analyzed using the TASSEL (Version 5.2.38) (Bradbury et al., 2007).

By analyzing *r*^2^ value of all pairs of SNPs located within 1 Mb of physical distance, the LD decay trend was found following the regression of negative natural logarithm. Heterozygosis, linkage disequilibrium decade, and kinship plot were generated using GAPIT (Lipka et al., 2012) with default parameters. For kinship plot, a heat map of the values in the values in the kinship matrix is created. Kinship matrix was using the VanRaden kinship algorithm (Tang et al., 2016).

GWAS was conducted the Fixed and random model Circulating Probability Unification (FarmCPU; Liu X. L. et al., 2016) with Bonferroni-corrected threshold with 0.01. This recently developed model selection algorithm takes into account the confounding problem between covariates and test marker by using both Fixed Effect Model (FEM) and a Random Effect Model (REM) (Arora et al., 2017). The first three principal components calculated using GAPIT were used as covariates. The quantile–quantile (Q–Q) plot was used for assessing how fit the model was to account for population structure.

## Result and discussion

### Polymorphic SNPs among the tested accessions

Of total 5,039 polymorphic SNP makers, 4,961 were mapped into 20 chromosome (Chr) and 31 scaffolds. Apart from unmapped 78 markers, 4,930 SNP markers were successfully mapped onto 20 chromosomes of the soybean genome (Gmax_275_Wm82.a2.v1; http://phytozome.jgi.doe.gov/pz/portal.html#!info?alias=Org_Gmax) using the stand-alone BLAST applications (BLAST+) (ftp://ftp.ncbi.nlm.nih.gov/blast/executables/blast+/LATEST) (Data Sheet 1, ftp://159.226.208.134/public/SNP_data.zip). In order to delimit the influence of batch specific or biased SNP markers on GWAS and population structure analysis, we deleted 459 batch specific or biased SNPs. The unbiased SNP was defined as the frequency of two homogenous nucleotide identities (e.g., AA, GG, or AG) at a given locus in a batch was 0.85 or higher. An unbiased marker having the same two nucleotide identities in two batches were kept for further analysis. According to this threshold of 0.85, 4,471 polymorphic SNP markers were enclosed for population structure and GWAS analysis (Data Sheet 1, ftp://159.226.208.134/public/SNP_data.zip).

**Figure 1:**
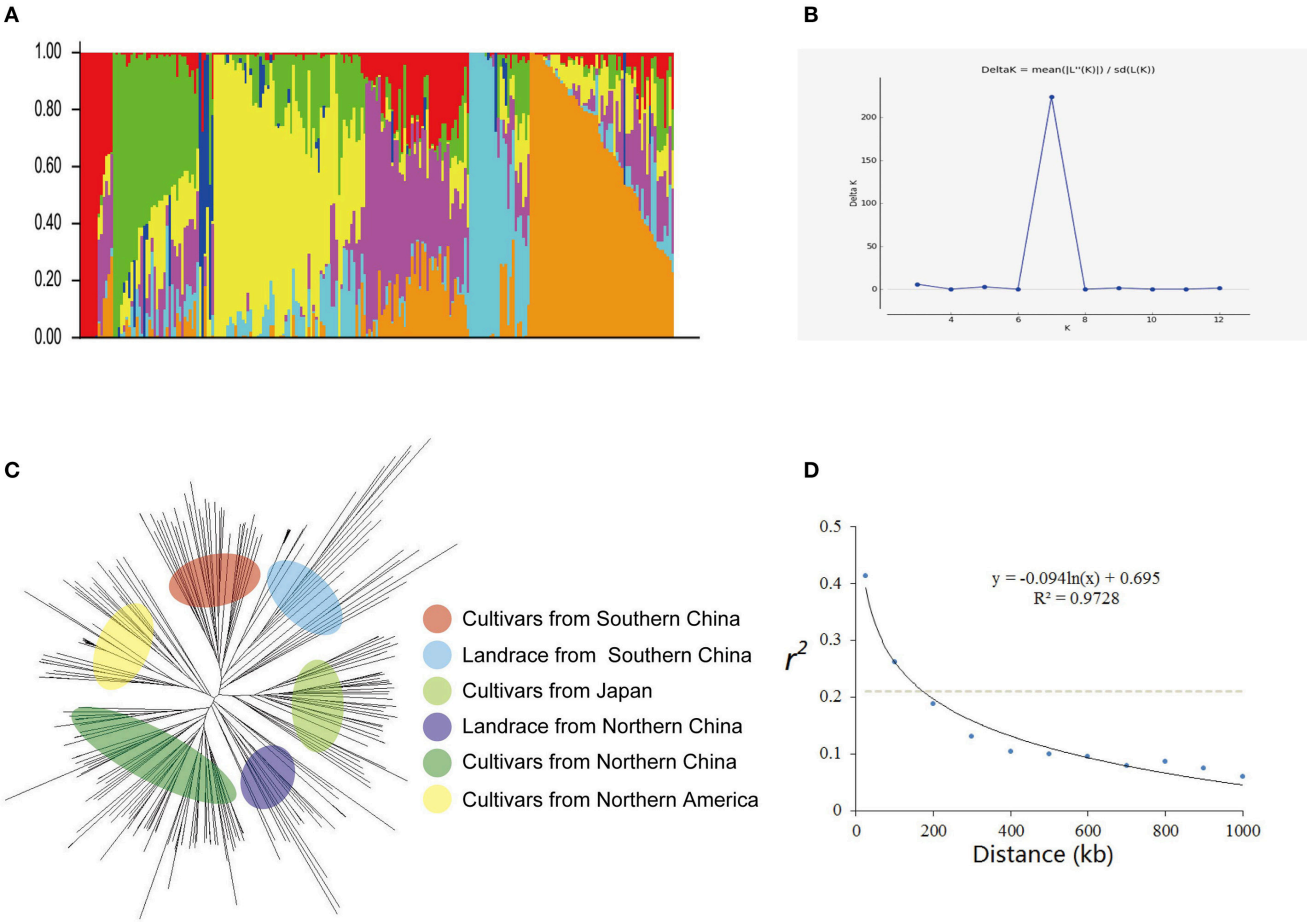
Genetic diversity and population structure of 235 soybean cultivars or accessions. **(A)** Population structure of 235 cultivars at K = 7. Each cultivar is represented by a single vertical line and color represents one cluster. **(B)** Estimated Delta K(probability of the data) calculated for K ranging from 2 to 12. **(C)** Phylogenetic tree constructed using neighbor-joining method. **(D)** Average linkage disequilibrium (LD) decay rate in the soybean genome. The mean LD decay rate was estimated as squared correlation coefficient (*r*^2^) using all pairs of SNPs located within 1 Mb of physical distance in a population of 235 soybean germplasm accessions. The dashed line in gray indicates the position where *r*^2^ dropped to half of its maximum value.

Rare SNPs other than two majority nucleotide identities were treated as unknown. Heterozygosis was calculated for both individuals and makers (Figure S1A). By analyzing *r*^2^ value of all pairs of SNPs located within 1 Mb of physical distance, the LD decay trend was found following the regression of negative natural logarithm (Figure 1D). The LD decay rate was estimated at 184 kb, where *r*^2^ drop to half of its maximum value (0.205). Also this trend was confirmed using GAPIT (Figure S1B). This LD rate calculated is well consistent with previous studies (Zhang et al., 2015; Song et al., 2016).

### Population structures

**Figure 2:**
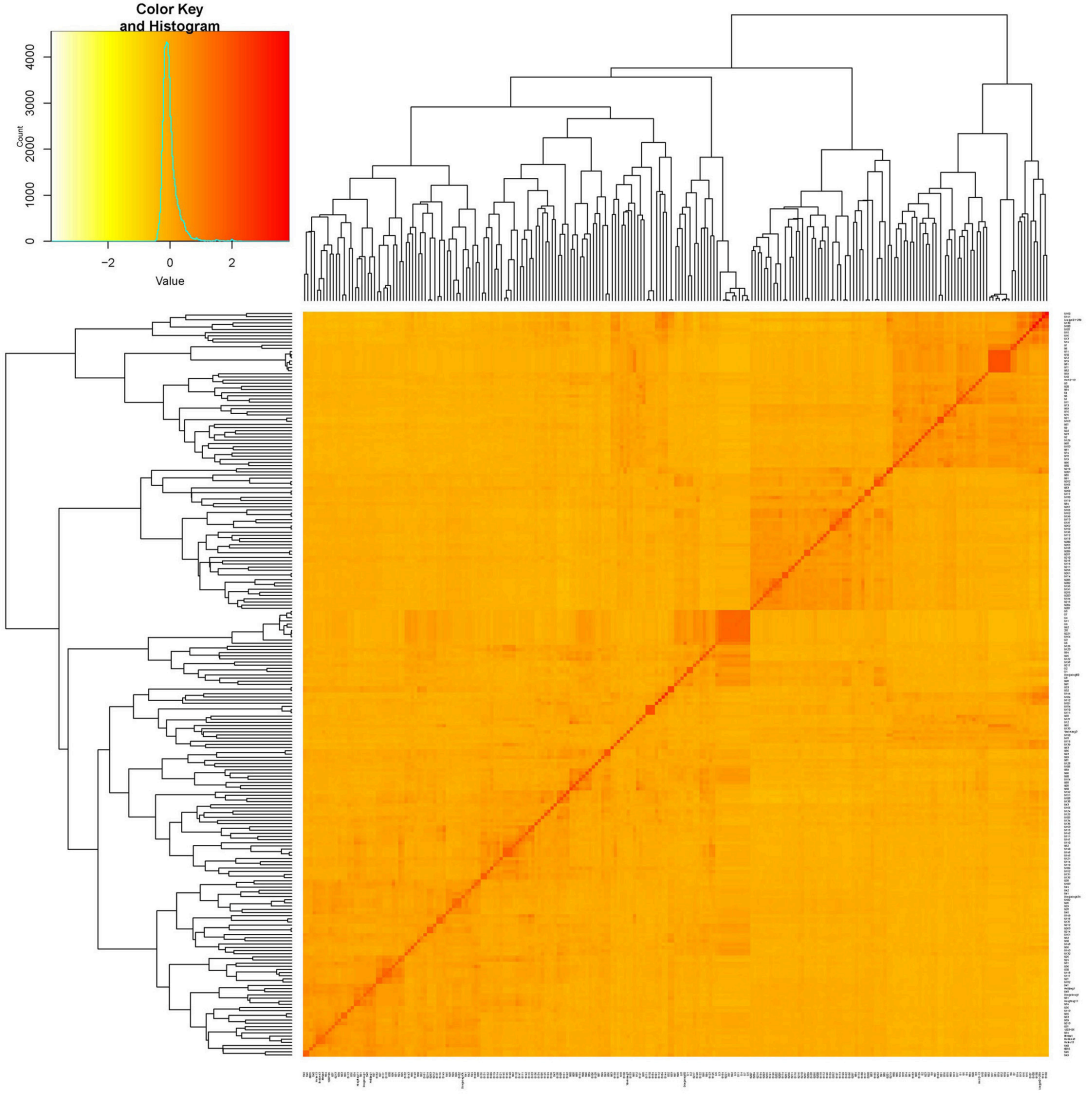
Kinship plot of 235 cultivars. The heat map of the values in the values in the kinship matrix was created using GAPIT (version 2).

Two hundred thirty five cultivars were originally obtained from different geographic origins, e.g., different latitudinal regions of China, Japan, USA. Apart from 5 landraces, the majority of set of germplasms are modern cultivars (Table S1). According to the population structure, the most likely value of K was 7 and such a portioning of the population was consistent with the significant delta K value (Figures 1A,B). Moreover, this result is also well in accordance with the neighbor- joining tree (Figure 1A). All cultivars are classified into 7 subgroups, which are generally in accordance with their geographic origins, Japan, Northern America, central China, Huang-huai region China, Northern area China, landraces (wild soybean) (Figure 1). This classification was also supported by the VanRaden kinship algorithm (Figure 2).

In this study, a relatively complex population structure (K = 7) was revealed in comparison of previous reports in which population structures (K = 2, 4, 9) were disclosed (Sonah et al., 2015; Liu Z. X. et al., 2016; Fang et al., 2017). After eliminating batch specific or biased markers, the set of 4471 markers might represents the core markers for this set of germplasm (Data Sheet 1, ftp://159.226.208.134/public/SNP_data.zip).

### GWAS on hilum color and seed coat color

**Figure 3:**
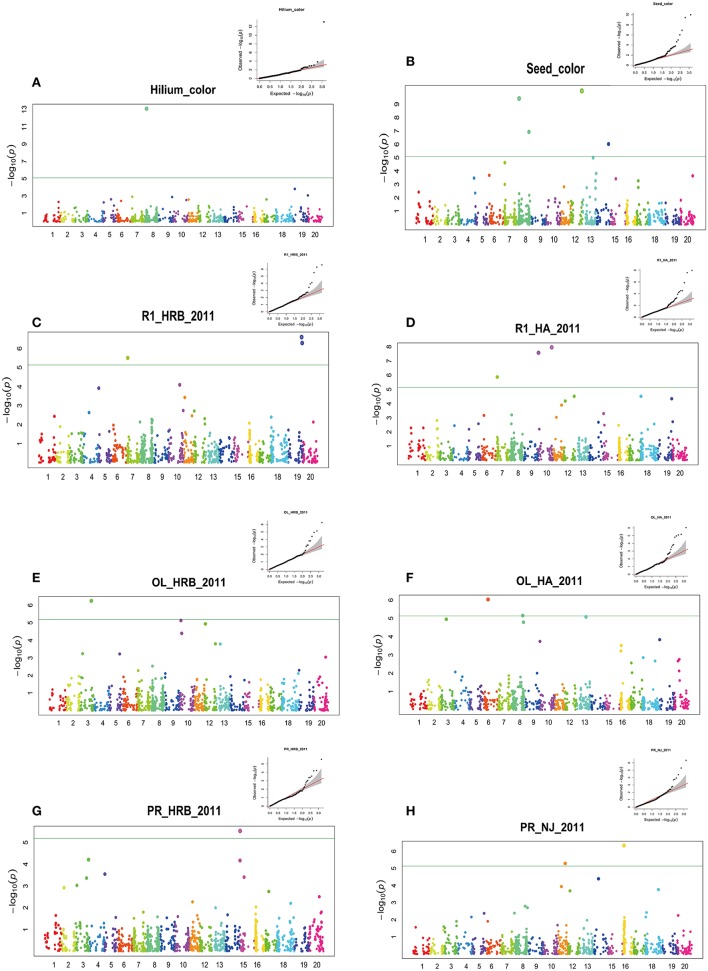
GWAS of seed hilum color, seed coat color, flowering time (R1), protein and oil using FarmCPU. Manhattan plots (bottom) and Quantile-quantile (upper right) plot. Negative log10 *P*-values from a genome-wide scan are plotted against SNP positions of 20 chromosomes. The horizontal dash line indicates the significant threshold (2 × 10^−5^). **(A)** Hilium color at Harbin in 2011; **(B)** Seed coat color at Harbin in 2011; **(C)** Flowering time (R1) at Harbin in 2011; **(D)** Flowering time (R1) at Huaian in 2011; **(E)** Oil content at Harbin in 2011; **(F)** Oil content at Huaian in 2011 **(G)** Protein content at Harbin in 2011 **(H)** Protein content at Nanjing in 2011.

Genetic control of seed hilum color has been well documented (Githiri et al., 2007; Oyoo et al., 2011; Cho et al., 2017). We used this trait as a control to monitor the accuracy of our GWAS analysis (Sonah et al., 2015). In this study, only one significant QTN peaked at Gm08_8571052_A_G-0_T_F_2177931718 (Chr08:8601055) was detected (Figure 3A, Table S2). *Chalcone synthase* (*CHS*) gene has been proved to regulate the hilum color. The significant QTN overlapped a *CHS* gene clustered region in chromosome 8 (Githiri et al., 2007; Oyoo et al., 2011; Fang et al., 2017). These CHS genes are *CHS5* (*Glyma.08G110400.1*, Chr08:8478834.8480215 reverse), *CHS3* (*Glyma.08G110900.1*, Chr08:8517799.8519303 reverse), *CHS4*(*Glyma.08G110500.1*, Chr08:8504479.8506020 reverse), *CHS3*(*Glyma.08G110300.1*, Chr08:8475793.8477410 forward), *CHS9*(*Glyma.08G109500.1*, Chr08:8397944.8399751 forward) (Cho et al., 2017).

We detected four significant QTNs for seed coat color using FarmCPU (Figure 3B). The major QTN was also located at 8622793 bp of chromosome 08. The major QTN detected for seed coat color was about 20 kb away from that for hilum (Figure 3). The clustered *CHS* family is considered to be candidate genes responsible for the seed coat color (Cho et al., 2017). Also other three QTNs were detected on chromosome 08 (41,212,762 bp), chromosome 12 (37,411,186 bp) and chromosome 14 (41,162,011 bp). A peak but not over the threshold was present on chromosome 13. Recently, seed coat bloom in wild soybeans is mainly controlled by Bloom1 (B1) on chromosome 13, which encodes a transmembrane transporter-like protein for biosynthesis of the bloom in pod endocarp (Zhang et al., 2018). Interestingly, this gene also elevated seed oil content in domesticated soybeans.

### GWAS on flowering time and maturity

**Figure 4:**
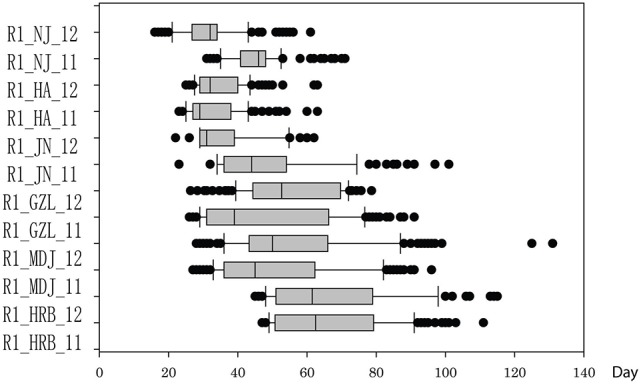
Phenotypic variations in flowering time (R1) of cultivars or accessions at different locations and in 2011 and 2012. The phenotypic segregation is shown in box-plot format. The interquartile region, median, and range are indicated by the box, the bold horizontal line, and the vertical line, respectively. For location, HRB, Harbin; MDJ, Mudanjiang; GZL, Gongzhuling; JN, Jinan; HA, Huaian; NJ, Nanjing. For years, 11, 2011; 12, 2012.

In this study, flowering time R1 and maturity R7 and R8 were evaluated in six geographic locations. For flowering time, the basic statistics of flowering time (R1) of cultivars were presented in Table 1. It took longer days to reach R1 in the northern locations, HRB, MDJ, and GZL (Figure 4). Other parameters such as Skewness, Kurtosis, K-S distance, K-S probability, SWilk W, SWilk probability indicated these traits were quantitatively inherited (Table 1). The correlation coefficients with a range of 0.592 to 0.978 between R1 of soybean cultivars grown at different locations in 2011 or 2012 (Table 2) were all statistically significant, which indicates this trait is genetically inherited, and also phenotypic data are validated.

**Table 1:** The basic statistics of flowering time (R1) of cultivars grown at different locations in 2011 or 2012.

	***N***	**Mean**	**Std dev**	**Std. error**	**Max**	**Min**	**Skewness**	**Kurtosis**	**K-S dist**.	**K-S Prob**.	**SWilk W**	**SWilk prob**
HRB_11	154	66.182	16.937	1.365	111.00	47.00	0.62	−0.87	0.17	<0.001	0.89	<0.001
HRB_12	156	66.622	19.244	1.541	115.00	45.00	0.89	−0.38	0.17	<0.001	0.87	<0.001
MDJ_11	158	51.076	17.882	1.423	96.00	27.00	0.78	−0.46	0.14	<0.001	0.91	<0.001
MDJ_12	164	54.848	18.49	1.444	131.00	28.00	1.39	2.23	0.20	<0.001	0.88	<0.001
GZL_11	150	46.84	18.684	1.526	91.00	26.00	0.77	−0.79	0.18	<0.001	0.86	<0.001
GZL_12	147	54.455	13.179	1.087	78.67	26.33	0.10	−1.27	0.14	<0.001	0.93	<0.001
JN_11	168	47.417	16.306	1.258	101.00	23.00	1.47	1.64	0.17	<0.001	0.83	<0.001
JN_12	150	36.053	10.031	0.819	62.00	22.00	1.28	0.51	0.26	<0.001	0.80	<0.001
HA_11	173	32.52	7.599	0.578	63.00	23.00	1.35	1.70	0.22	<0.001	0.85	<0.001
HA_12	174	34.529	7.338	0.556	63.00	25.00	1.22	1.45	0.18	<0.001	0.88	<0.001
NJ_11	174	45.546	8.302	0.629	71.00	31.00	0.93	1.51	0.22	<0.001	0.89	<0.001
NJ_12	174	31.489	8.796	0.667	61.00	16.00	0.87	1.17	0.16	<0.001	0.93	<0.001

*Name in the first column or the first row is composed of location, and year. For location, HRB, Harbin; MDJ, Mudanjiang; JN, Jinan; HA, Huaian; NJ, Najing. For years, 11, 2011; 12, 2012. For protein or oil contents, PR, protein content; OL, oil content*.

**Table 2:** The correlation coefficients between R1 (first flower) of soybean cultivars grown at different locations in 2011 or 2012.

	**HRB_11**	**HRB_12**	**MDJ_11**	**MDJ_12**	**GZL_11**	**GZL_12**	**JN_11**	**JN_12**	**HA_11**	**HA_12**	**NJ_11**	**NJ_12**
HRB_11		0.928^**^	0.768^**^	0.744^**^	0.878^**^	0.797^**^	0.753^**^	0.769^**^	0.870^**^	0.873^**^	0.648^**^	0.616^**^
HRB_12	0.928^**^		0.808^**^	0.793^**^	0.914^**^	0.780^**^	0.791^**^	0.882^**^	0.911^**^	0.913^**^	0.665^**^	0.625^**^
MDJ_11	0.768^**^	0.808^**^		0.888^**^	0.789^**^	0.685^**^	0.762^**^	0.795^**^	0.830^**^	0.827^**^	0.735^**^	0.697^**^
MDJ_12	0.744^**^	0.793^**^	0.888^**^		0.825^**^	0.665^**^	0.830^**^	0.871^**^	0.863^**^	0.858^**^	0.789^**^	0.758^**^
GZL_11	0.878^**^	0.914^**^	0.789^**^	0.825^**^		0.795^**^	0.797^**^	0.847^**^	0.885^**^	0.884^**^	0.699^**^	0.695^**^
GZL_12	0.797^**^	0.780^**^	0.685^**^	0.665^**^	0.795^**^		0.592^**^	0.578^**^	0.708^**^	0.723^**^	0.530^**^	0.482^**^
JN_11	0.753^**^	0.791^**^	0.762^**^	0.830^**^	0.797^**^	0.592^**^		0.877^**^	0.886^**^	0.890^**^	0.751^**^	0.703^**^
JN_12	0.769^**^	0.882^**^	0.795^**^	0.871^**^	0.847^**^	0.578^**^	0.877^**^		0.897^**^	0.896^**^	0.698^**^	0.698^**^
HA_11	0.870^**^	0.911^**^	0.830^**^	0.863^**^	0.885^**^	0.708^**^	0.886^**^	0.897^**^		0.978^**^	0.796^**^	0.768^**^
HA_12	0.873^**^	0.913^**^	0.827^**^	0.858^**^	0.884^**^	0.723^**^	0.890^**^	0.896^**^	0.978^**^		0.791^**^	0.768^**^
NJ_11	0.648^**^	0.665^**^	0.735^**^	0.789^**^	0.699^**^	0.530^**^	0.751^**^	0.698^**^	0.796^**^	0.791^**^		0.935^**^
NJ_12	0.616^**^	0.625^**^	0.697^**^	0.758^**^	0.695^**^	0.482^**^	0.703^**^	0.698^**^	0.768^**^	0.768^**^	0.935^**^	

*Name in the first column or the first row is composed of triat, location, and year. For location, HRB, Harbin; MDJ, Mudanjiang; JN, Jinan; HA, Huaian; NJ, Najing. For years, 11, 2011; 12, 2012. For protein or oil contents, R1, from emergence to first flower*.

** , Correlation coefficient is statistically highly significant (P < 0.01);

^*^, Correlation coefficient is statistically significant (P < 0.05).

Statistical analysis (Table 3) showed that broad sense heritability was 0.5833.

**Table 3:** The heritability estimates were calculated using variance components obtained by lme4 of R package.

**Groups**	**Variance**	**Std. dev**.	**F**	**Heritability**
**STASTICAL ANALYSIS FOR FLOWERING TIME (R1)**				
Cultivar^*^YEAR	0.9737	0.9868	0.4869	
Cultivar^*^LOC	244.9000	15.6500	48.9800	
Cultivar	72.8300	8.5340		
YEAR	0.0000	0.0000	0.0000	
REP in LOC^*^YEAR	2.6090	1.6150	0.2609	
LOC	47.4000	6.8840		
Residual	23.0200	4.7980	2.3020	
				0.5833
**Groups name**	**Variance**	**Std. dev**.	**F**	**Heritability**
**STASTICAL ANALYSIS FOR OIL CONTENT (OL)**				
Cultivar^*^YEAR	0.3205	0.5661	0.16025	
Cultivar^*^LOC	2.8742	1.6953	0.57484	
Cultivar	1.4405	1.2002		
YEAR	0.1168	0.3417	0.0584	
REP in LOC^*^YEAR	0.1153	0.3396	0.01153	
LOC	0.1414	0.376		
Residual	0.7645	0.8744	0.07645	
				0.6364
Cultivar^*^YEAR	**Variance**	**Std. dev**.	**F**	**Heritability**
**STASTICAL ANALYSIS FOR PROTEIN CONTENT (PR)**				
Cultivar^*^LOC	3.11	1.7635	1.555	
Cultivar	1.6388	1.2801	0.32776	
YEAR	1.6875	1.299		
REP in LOC^*^YEAR	0.4832	0.6951	0.2416	
LOC	4.6175	2.1488	0.46175	
Residual	1.0955	1.0466		
Residual	2.4393	1.5618	0.24393	
				0.3947

Although phenotypic data for R7 and R8 were not conducted in all locations, the basic distributions were presented in Figure S2, which was similar to R1 trait. Since some cultivars could not reached R7 or R8 before frost in northern locations, HRB, MDJ, and GZL.

In order to analyze the relationship between R1 and R7/R8, the correlation coefficients matrix were generated and listed in Table S2. The correlation coefficients of R7 (R8) between different geographic locations or years were statistically significant except for that between MDJ and southern location, HA and NJ. The correlation coefficients between R1 and R7 or R8 were higher in the same location than in different location. Considering maturity genes, such as *E1*–*E4*, are controlling flowering time as well as maturity, we also enclosed R7 and R8 for GWAS.

Although no consistent QTNs for flowering time and maturity were identified across all environments, a total of 30 consistent QTNs were detected for flowering time (R1) or maturity (R7 and R8) on 16 chromosomes (Figures 3C–H; Table 4; Figures S3–S6; Table S3). In Table 4 and Table S3, we only listed the QTN that has been detected more than three environments. In Table 4, we listed the corresponding QTLs listed in SoyBase or known genes with a physical distance less than 5 Mb.

**Table 4:** Physical position, *P*-value, effect, and distance to known QTL or known genes of QTN for flowering time (R1) and maturity (R7 and R8) detected using FarmCPU.

**Chr**	**Position**	**LG**	**Average of *P*. value**	**Average of effect**	**Distance to known QTL or gene (Kb)**	**QTL in SoyBase or known gene**
3	1094352	N	4.05 × 10^−3^	−2.38	4,570	Pod maturity19-3 (Guzman et al., 2007)
4	6130517	C1	4.36 × 10^−3^	2.94	266	Pod maturity 1-1 (Keim et al., 1990)
4	36583411	C1	1.78 × 10^−3^	−2.66		
4	39484122	C1	4.23 × 10^−3^	0.46		
6	10919417	C2	1.29 × 10^−3^	2.47	2,130	Pod maturity13-3 (Specht et al., 2001)
7	4918268	M	2.20 × 10^−6^	−5.99	92	First flower 2-2 (Mansur et al., 1993).
7	4928246	M	4.40 × 10^−6^	8.22	82.45	First flower 2-2 (Mansur et al., 1993)
7	8251563	M	3.16 × 10^−3^	4.01	2,260	First flower 6-2 (Orf et al., 1999)
8	18036672	A2	3.92 × 10^−3^	3.74		
9	49446558	K	1.02 × 10^−3^	−2.07	4,730	First flower 24-4 (Kuroda et al., 2013)
10	45054578	O	7.56 × 10^−6^	7.40	240	E2 (Watanabe et al., 2011)
11	10752436	B1	2.97 × 10^−4^	3.66	83.7	First flower 11-2 (Gai et al., 2007)
11	28002694	B1	2.70 × 10^−3^	2.42	966	First flower 8-4 (Yamanaka et al., 2001)
12	37271658	H	9.74 × 10^−4^	3.74^$^	535	Pod maturity 37-3 (Panthee et al., 2007)
14	5766604	B2	8.81 × 10^−4^	5.52		
14	44255110	B2	2.98 × 10^−4^	−4.11	540	First flower 21-1 (Reinprecht et al., 2006)
15	1348441	E	1.22 × 10^−3^	−4.14	1,170	Pod maturity 34-4 (Yao et al., 2015)
16	2643365	J	3.94 × 10^−3^	−1.58	995	Pod maturity 19-6 (Guzman et al., 2007)
16	3623089	J	4.29 × 10^−3^	3.09	89	GmFT5a (Takeshima et al., 2016)
17	5422636	D2	4.14 × 10^−3^	−2.19		
18	1883973	G	2.18 × 10^−5^	−4.97	87.5	First flower 21-4 (Reinprecht et al., 2006)
18	3737376	G	3.52 × 10^−3^	2.42	3,290	Pod maturity 16-2 (Kabelka et al., 2004)
18	24606904	G	3.03 × 10^−3^	2.44	2,230	Pod maturity 34-5 (Yao et al., 2015)
18	45935966	G	3.68 × 10^−3^	−3.09	3,240	First flower 10-2 (Tasma et al., 2001)
19	35744249	L	9.82 × 10^−6^	−4.16	1,440	First flower 15-2 (Komatsu et al., 2007)
19	44839670	L	2.48 × 10^−3^	2.17	343	First flower 2-3 (Mansur et al., 1993)
19	46634511	L	2.84 × 10^−3^	−3.21	125	Pod maturity 4-3 (Mansur et al., 1996); First
					406	flower 16-4 (Khan et al., 2008)
19	46730237	L	2.77 × 10^−3^	0.27^&^	437	E3 (Watanabe et al., 2009)
20	36021032	I	4.61 × 10^−4^	2.48	821	E4 (Liu et al., 2008)

*Only QTN that was detected more than three environments were listed*.

$ Effect of−3.524 for R1_MDJ_2012 was not counted due to the oppositing effect;

& *effect of−6.267737 for R1_GZL_2011 was not counted due to the oppositing effect*.

In chromose 10 (LG O), we detected a QTN at 45054578 with effect of 7.40 (Table 4; Table S3), which is about 240 kb away from the reported E2 gene (Watanabe et al., 2009). This gene is a major genetic factor controlling flowering time, maturity, geographic adaption in Chinese cultivars (Zhai et al., 2014a; Wang et al., 2016; Fang et al., 2017; Langewisch et al., 2017). In chromosome 19 (LG L), 4 QTN were detected to be significantly associated with flowering time or maturity (Table 4; Table S3). Three QTN at 44839670, 46634511, 46730237 were detected in 5, 12, and 5 environments respectively. QTN at position of 44839670 on chromosome 19 exhibited consistent effect on flowering time or maturity with average of 2.17 day. QTN at 46634511, displayed homogeneous effect on flowering or maturity with average of −3.21 days. In this region, *E3* gene, encoding phytochrome A (PHYA), is located from 47633059 to 47641958. The QTN (Gm19_46611973_C_T-1_B_F_2179344248) at 46730237 were detected having four location with positive (suppressing flowering) effect (average of, while in QTN for R1 in GZL in 2011 displayed an oppositing effect of −6.27 days. In generally, the E3 region is strongly associated with flowering time and domestication (Watanabe et al., 2009; Zhai et al., 2014a; Zhou et al., 2015; Langewisch et al., 2017). The QTN disclosed in this study might this region is very important in term of regulation of flowering time or maturity. However, the authenticity of these QTNs or the relationship with the *E3* gene merits further investigation.

On chromosome 6, a QTN (Gm06_10891060_T_C-1_B_F_2179335984) was detected at 10919417 with effect of 2.47 day. The *E1* gene is located in the pericentromeric region from 20207253 to 20207829 (Xia et al., 2012b) of chromosome 6. Glyma.06G207800.1 in phytozome is physically corresponding to the *E1* gene, however, this coding region of this gene was annotated from 20207077 to 2020794. The lack of polymorphic SNP in the *E1* region might account for not being able to detect this major gene. Another Phytochrome A gene, *E4*, located at Chr20:33236018.33241692 (forward), was reported to be less diversified among Chinese and American cultivars (Zhai et al., 2014b; Langewisch et al., 2017). A QTN (Gm20_34881595_C_T-1_B_F_2179344630) was detected about 3 Mb away from *E4* gene. *GmFT5a*, an FT homolog, located at Chr16:4135885.4137742 (reverse) about 89 kb from the QTN (Gm16_3598173_C_T-1_B_F_2179342018 with average effect of 3.09) detected (Table 4; Table S3). Other QTNs detected over 3 environments were mapped on chromosome 3, 4, 7, 8, 9, 11, 12, 14, 15, 16, 17, 18, 19 (Figures 3C–H; Table 4; Figures S3–S6; Table S3). Among them, QTN (Gm11_10721006_A_G-1_T_F_2179339194) at 10752436 bp on Chr 11 (LG B1), QTN (Gm12_37315664_A_G-1_T_F_2179339946) at 37271658 on Chr 12 (LG H), QTN(Gm15_1349135_T_C-1_B_F_2179341354) at 1348441 of on Chr 15 (LG E); QTL (Gm18_34401760_G_A-1_T_F_2179343324) at 24606904 on Chr 18 (LG G) were identified in 7 or more environments. Fang et al. (2017) also reported a QTN on chromosome 18 (Fang et al., 2017), whether QTN (Gm18_34401760_G_A-1_T_F_2179343324) is the same as the QTLs reported by other researchers (SoyBase, www.soybase.org) merits further investigation.

In our previous study, the genotypes at *E1, E2, E3*, and *E4* of 180 cultivars revealed great allelic variations at *E1* and *E3* genes (Zhai et al., 2014b). The power of GWAS to capture a certain trait often depends on the frequency of the accessions with contrast phenotypic value in the population being investigated (Yan et al., 2017). In the previous GWAS studies, fewer QTNs were detected for this trait. When the modern cultivars only a QTN corresponding to *E3* was detected at a natural population of 304 short-season soybean lines (K = 9) (Sonah et al., 2015). While using 892 cultivars (K = 4), only a QTN corresponds to *E2* locus was identified (Fang et al., 2017).

No universal QTN was detected over all environments in this study. Common QTNs detected in three or more environments are also informative for us to understand this trait, although authenticity of these QTNs detected in this study need to be verified. GWAS and biparental linkage mapping are commentary each other in mapping and thereafter gene cloning. At present, around 50 biparental populations were generated using the cultivars in this study. We will use these populations to verify the QTN obtained in this study. Fine-mapping or positional cloning will be performed when a novel gene or QTN is verified.

### GWAS of protein and oil contents of cultivar seeds

**Figure 5:**
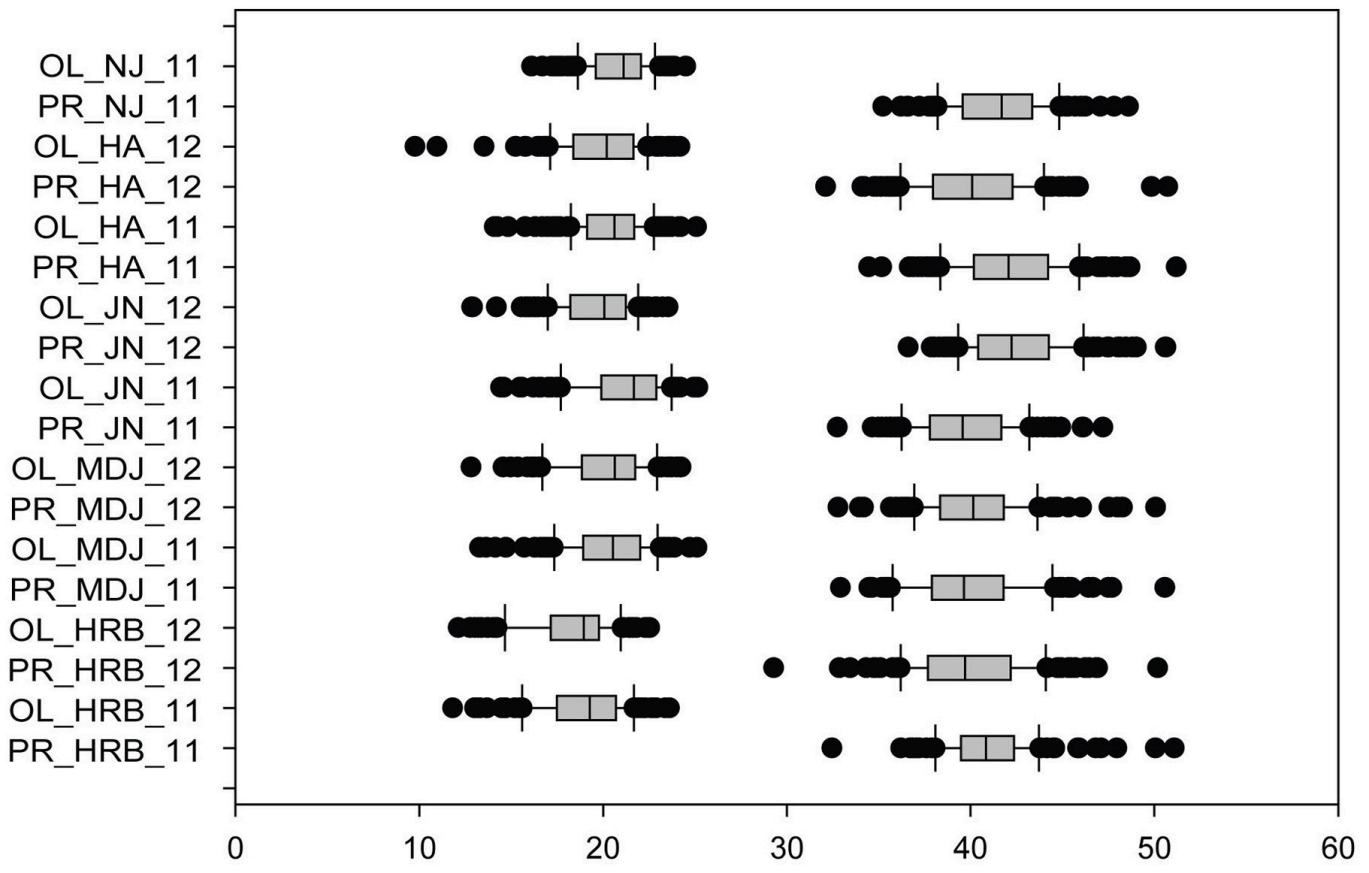
Phenotypic variations in protein (PR) and oil (OL) contents of cultivars or accessions at different locations and in 2011 and 2012. The phenotypic segregation is shown in box-plot format. The interquartile region, median, and range are indicated by the box, the bold horizontal line, and the vertical line, respectively. For location, HRB, Harbin; MDJ, Mudanjiang; GZL, Gongzhuling; JN, Jinan; HA, Huaian; NJ, Nanjing. For years, 11, 2011; 12, 2012.

In this study, protein and oil contents were simultaneously measured in 5 geographic location in 2011 and 2012. The basic statistics of two traits were listed in Table 5 and presented in Figure 5. The parameters such as Skewness, Kurtosis, K-S distance, K-S probability, SWilk W, SWilk probability indicated this trait were quantitatively inherited (Table 5). The correlation coefficients between protein and oil were presented in Table 6. From the correlation coefficients, the protein contents were negatively and significantly correlated to oil content in the same environments or different environments; while the protein contents in an environments was positively correlated to protein contents in other environments (Table 6, Figure 5). The trend was the same for oil contents. According to statistical analysis, the broad sense heritability for oil and protein were 0.6364 and 0.3947. When we used data for protein and oil contents obtained in 9 environments for GWAS using FarmCPU, 16 consistent QTNs for protein and oil contents were detected for oil or protein over 3 environments (Table 7; Table S4; Figures 3G,H, Figures S7, S8). Eleven QTNs were detected having antagonistic effects on protein and oil content, while 4 QTNs soly for oil content, and one QTN soly for protein content. Of eleven QTN for both traits detected over 3 environments, each QTN showed antagonistic effects on protein and oil contents, which indicated these QTNs are involved in biological pathway affecting both oil and protein. Major QTL were repeatedly detected on Chromosome 20 (LG I) and 15 (LG E) using America cultivars (Patil et al., 2017). In this study, we detected three QTNs on Chromosome 20 (LG O). Two QTNs were identified for both traits, QTN (Gm20_2372509_T_C-1_T_R_2179344425) at position of 2366428 with antagonistic effects on protein (0.431691) and oil (-0.45203) and QTN (Gm20_7927513_A_G-1_T_F_2179344472) with antagonistic effects on protein(0.76146) and oil (-0.47998). Another QTN (Gm20_38151772_C_T-1_T_R_2179344711) for oil with effect of−0.53353 was identified on chromosome 20. We did not detect any consistent QTN on Chr 15 (LG E). All 16 QTNs mapped in this study (Table 7) were physically near (less than 5 Mb) QTL reported in SoyBase.

**Table 5:** The basic statistics of protein and oil contents of cultivars grown at different locations in 2011 or 2012.

	***N***	**Mean**	**Std dev**	**Std. error**	**Max**	**Min**	**Skewness**	**Kurtosis**	**K-S dist**.	**K-S Prob**.	**SWilk W**	**SWilk prob**
PR_HRB_11	143	40.979	2.59	0.217	51.08	32.47	0.61	2.43	0.06	0.145	0.96	<0.001
OL_HRB_11	143	18.952	2.325	0.194	23.61	11.83	−0.55	0.05	0.07	0.103	0.98	0.016
PR_HRB_12	145	39.955	3.297	0.274	50.18	29.28	0.10	0.41	0.05	0.391	0.99	0.673
OL_HRB_12	145	18.347	2.26	0.188	22.53	12.13	−0.74	0.12	0.12	<0.001	0.95	<0.001
PR_MDJ_11	126	39.938	3.184	0.284	50.57	32.93	0.55	0.37	0.08	0.033	0.98	0.025
OL_MDJ_11	126	20.334	2.262	0.201	25.11	13.28	−0.67	0.54	0.09	0.01	0.97	0.006
PR_MDJ_12	129	40.299	2.789	0.246	50.06	32.79	0.51	1.38	0.07	0.164	0.98	0.018
OL_MDJ_12	129	20.184	2.28	0.201	24.24	12.82	−0.66	0.06	0.09	0.015	0.96	0.001
PR_JN_11	140	39.679	2.672	0.226	47.21	32.75	0.20	−0.21	0.04	0.653	0.99	0.712
OL_JN_11	140	21.187	2.31	0.195	25.16	14.44	−0.74	0.14	0.10	<0.001	0.96	<0.001
PR_JN_12	150	42.474	2.717	0.222	50.63	36.61	0.60	0.19	0.07	0.109	0.98	0.008
OL_JN_12	150	19.612	2.033	0.166	23.54	12.86	−0.71	0.45	0.10	0.001	0.96	<0.001
PR_HA_11	164	42.222	2.949	0.23	51.19	34.47	0.14	−0.08	0.04	0.649	1.00	0.953
OL_HA_11	164	20.393	1.918	0.15	25.09	14.09	−0.55	0.84	0.05	0.273	0.98	0.011
PR_HA_12	168	40.091	3.002	0.232	50.72	32.13	0.33	0.52	0.04	0.651	0.99	0.175
OL_HA_12	168	19.928	2.298	0.177	24.18	9.78	−1.02	2.44	0.07	0.066	0.95	<0.001
PR_NJ_11	159	41.598	2.523	0.2	48.59	35.23	0.06	−0.29	0.06	0.264	0.99	0.478
OL_NJ_11	159	20.867	1.676	0.133	24.51	16.11	−0.36	−0.29	0.07	0.039	0.99	0.091

*Name in the first column or the first row is composed of tran, location, and year. For trait, PR, protein content; OL, oil content; For location, HRB, Harbin; MDJ, Mudanjiang; JN, Jinan; HA, Huaian; NJ, Najing. For years, 11, 2011; 12, 2012. For protein or oil contents, PR, protein content; OL, oil content*.

**Table 6:** The correlation coefficients between seed protein content and oil content of soybean cultivars grown at different locations in 2011 or 2012.

	**PR_HRB_11**	**OL_HRB_11**	**PR_HRB_12**	**OL_HRB_12**	**PR_MDJ_11**	**OL_MDJ_11**	**PR_MDJ_12**	**OL_MDJ_12**	**PR_JN_11**	**OL_JN_11**	**PR_JN_12**	**OL_JN_12**	**PR_HA_11**	**OL_HA_11**	**PR_HA_12**	**OL_HA_12**	**PR_NJ_11**	**OL_NJ_11**
PR_HRB_11		−0.373^**^	0.358^**^	−0.208^*^	0.676^**^	−0.502^**^	0.507^**^	−0.330^**^	0.738^**^	−0.447^**^	0.624^**^	−0.462^**^	0.477^**^	−0.499^**^	0.442^**^	−0.534^**^	0.395^**^	−0.351^**^
OL_HRB_11	−0.373^**^		−0.565^**^	0.789^**^	−0.470^**^	0.790^**^	−0.523^**^	0.760^**^	−0.697^**^	0.860^**^	−0.377^**^	0.638^**^	−0.470^**^	0.631^**^	−0.446^**^	0.648^**^	−0.474^**^	0.660^**^
PR_HRB_12	0.358^**^	−0.565^**^		−0.754^**^	0.537^**^	−0.540^**^	0.423^**^	−0.474^**^	0.577^**^	−0.532^**^	0.416^**^	−0.366^**^	0.386^**^	−0.431^**^	0.475^**^	−0.491^**^	0.525^**^	−0.438^**^
OL_HRB_12	−0.208^*^	0.789^**^	−0.754^**^		−0.429^**^	0.722^**^	−0.495^**^	0.746^**^	−0.591^**^	0.759^**^	−0.352^**^	0.537^**^	−0.478^**^	0.632^**^	−0.514^**^	0.630^**^	−0.423^**^	0.590^**^
PR_MDJ_11	0.676^**^	−0.470^**^	0.537^**^	−0.429^**^		−0.679^**^	0.487^**^	−0.390^**^	0.605^**^	−0.482^**^	0.455^**^	−0.483^**^	0.336^**^	−0.394^**^	0.288^**^	−0.365^**^	0.332^**^	−0.360^**^
OL_MDJ_11	−0.502^**^	0.790^**^	−0.540^**^	0.722^**^	−0.679^**^		−0.549^**^	0.716^**^	−0.642^**^	0.787^**^	−0.420^**^	0.688^**^	−0.441^**^	0.605^**^	−0.374^**^	0.588^**^	−0.450^**^	0.606^**^
PR_MDJ_12	0.507^**^	−0.523^**^	0.423^**^	−0.495^**^	0.487^**^	−0.549^**^		−0.754^**^	0.658^**^	−0.643^**^	0.570^**^	−0.609^**^	0.568^**^	−0.549^**^	0.432^**^	−0.467^**^	0.526^**^	−0.535^**^
OL_MDJ_12	−0.330^**^	0.760^**^	−0.474^**^	0.746^**^	−0.390^**^	0.716^**^	−0.754^**^		−0.620^**^	0.813^**^	−0.443^**^	0.706^**^	−0.576^**^	0.652^**^	−0.442^**^	0.536^**^	−0.507^**^	0.663^**^
PR_JN_11	0.738^**^	−0.697^**^	0.577^**^	−0.591^**^	0.605^**^	−0.642^**^	0.658^**^	−0.620^**^		−0.778^**^	0.718^**^	−0.746^**^	0.659^**^	−0.656^**^	0.558^**^	−0.661^**^	0.651^**^	−0.677^**^
OL_JN_11	−0.447^**^	0.860^**^	−0.532^**^	0.759^**^	−0.482^**^	0.787^**^	−0.643^**^	0.813^**^	−0.778^**^		−0.535^**^	0.870^**^	−0.578^**^	0.689^**^	−0.455^**^	0.677^**^	−0.611^**^	0.717^**^
PR_JN_12	0.624^**^	−0.377^**^	0.416^**^	−0.352^**^	0.455^**^	−0.420^**^	0.570^**^	−0.443^**^	0.718^**^	−0.535^**^		−0.720^**^	0.569^**^	−0.534^**^	0.450^**^	−0.491^**^	0.532^**^	−0.516^**^
OL_JN_12	−0.462^**^	0.638^**^	−0.366^**^	0.537^**^	−0.483^**^	0.688^**^	−0.609^**^	0.706^**^	−0.746^**^	0.870^**^	−0.720^**^		−0.571^**^	0.665^**^	−0.405^**^	0.576^**^	−0.587^**^	0.693^**^
PR_HA_11	0.477^**^	−0.470^**^	0.386^**^	−0.478^**^	0.336^**^	−0.441^**^	0.568^**^	−0.576^**^	0.659^**^	−0.578^**^	0.569^**^	−0.571^**^		−0.753^**^	0.667^**^	−0.638^**^	0.661^**^	−0.576^**^
OL_HA_11	−0.499^**^	0.631^**^	−0.431^**^	0.632^**^	−0.394^**^	0.605^**^	−0.549^**^	0.652^**^	−0.656^**^	0.689^**^	−0.534^**^	0.665^**^	−0.753^**^		−0.657^**^	0.784^**^	−0.544^**^	0.715^**^
PR_HA_12	0.442^**^	−0.446^**^	0.475^**^	−0.514^**^	0.288^**^	−0.374^**^	0.432^**^	−0.442^**^	0.558^**^	−0.455^**^	0.450^**^	−0.405^**^	0.667^**^	−0.657^**^		−0.830^**^	0.519^**^	−0.491^**^
OL_HA_12	−0.534^**^	0.648^**^	−0.491^**^	0.630^**^	−0.365^**^	0.588^**^	−0.467^**^	0.536^**^	−0.661^**^	0.677^**^	−0.491^**^	0.576^**^	−0.638^**^	0.784^**^	−0.830^**^		−0.552^**^	0.669^**^
PR_NJ_11	0.395^**^	−0.474^**^	0.525^**^	−0.423^**^	0.332^**^	−0.450^**^	0.526^**^	−0.507^**^	0.651^**^	−0.611^**^	0.532^**^	−0.587^**^	0.661^**^	−0.544^**^	0.519^**^	−0.552^**^		−0.726^**^
OL_NJ_11	−0.351^**^	0.660^**^	−0.438^**^	0.590^**^	−0.360^**^	0.606^**^	−0.535^**^	0.663^**^	−0.677^**^	0.717^**^	−0.516^**^	0.693^**^	−0.576^**^	0.715^**^	−0.491^**^	0.669^**^	−0.726^**^	

*Name in the first column or the first row is composed of triat, location, and year. For location, HRB, Harbin; MDJ, Mudanjiang; JN, Jinan; HA, Huaian; NJ, Najing. For years, 11, 2011; 12, 2012. For protein or oil contents, PR, protein content; OL, oil content*.

** , Correlation coefficient is statistically highly significant (P < 0.01);

* *, Correlation coefficient is statistically significant (P < 0.05)*.

**Table 7:** Physical position, *P-*value, effect, and distance to known QTL or known genes of QTN for protein and oil content (PR/OL), oil content only (OL) and protein content only (PR) using FarmCPU.

**Trait**	**Chr**	**LG**	**Position**	***P*-value**	**Effect on PR**	**Effect on OL**	**Distance to known QTL or gene**	**QTL information from SoyBase**
PR/OL	1	D1a	8869097	0.002549	0.00955	−0.62331	1,140	Seed protein 3-5 (Brummer et al., 1997)
							1,140	Seed oil 42-20 (Han et al., 2015)
	5	A1	37361373	0.002501	−0.5009	0.387996	2,900	Seed protein 41-1(Jun et al., 2008)
							346	Seed oil 4-2 (Brummer et al., 1997)
	8	A2	8613057	0.001182	2.506472	−1.08523	17	Seed protein 26-1 (Reinprecht et al., 2006)
							579	Seed oil 30-3 (Liang et al., 2010)
	13	F	13865497	0.000118	1.287005	−0.82343	753	Seed protein 36-22 (Mao et al., 2013)
							1,441	Seed oil 24-4 (Qi et al., 2011)
	16	J	4582681	0.003177	1.316393	−0.75341	382	Seed protein 4-7 (Lee et al., 1996)
							370	Seed oil 43-20 (Mao et al., 2013)
	17	D2	11939572	0.002254	1.1395	−0.50493	302	Seed protein 37-6 (Wang et al., 2014)
							570	Seed Oil-011 (Qi et al., 2011)
	18	G	3737376	0.004217	0.477983	−0.31451	111	Seed protein 20-1 (Panthee et al., 2005)
							1,431	Seed oil 42-31 (Han et al., 2015)
	18	G	43143230	0.000556	1.004969	−0.44686		
							1,612	Seed oil 42-33 (Han et al., 2015)
	19	L	809351	0.002534	−0.93037	0.502982	34	Seed protein 41-8 (Jun et al., 2008)
							423	Seed oil 43-27 (Mao et al., 2013)
	20	I	2366428	0.003448	0.431691	−0.45203	319	Seed protein 26-4 (Reinprecht et al., 2006)
							319	Seed oil 14-3 (Csanádi et al., 2001)
	20	I	20469935	0.002656	0.76146	−0.47998	3,710	Seed protein 1-2 (Diers et al., 1992)
							3,708	Seed oil 2-2 (Csanádi et al., 2001)
PR	5	A1	37987063	0.002457	0.621571	–	1,850	Seed protein-011 (Pathan et al., 2013)
OL	7	M	8251563	0.000152	–	−0.87557	31	Seed oil 23-6 (Hyten et al., 2004)
	8	A2	3823489	0.00048	–	0.420013	1,949	Seed oil 24-1 (Qi et al., 2011)
	11	B1	10752436	0.001861	–	−0.77553	749	Seed oil 39-2 (Wang et al., 2014)
	20	I	39264676	0.002104	–	−0.53353	1,002	Seed oil 42-39 (Han et al., 2015)

*Only QTN that was detected more than three environments were listed*.

### Conclusion and further consideration

Instead of traditional molecular markers, e.g., SSR, AFLP, advances in sequencing technologies have enabled high-density array and GBS to be widely applied to genomic and genetic study to dissect genetic population structure and GWAS (Sonah et al., 2013; Bandillo et al., 2015; Wen et al., 2015; Zhang et al., 2015; Contreras-Soto et al., 2017; Fang et al., 2017; Yan et al., 2017). However, this study employed a medium density array to reveal population genetic structure, the result showed the quality of the population genetic study has been improved by elimination of some batch specific or biased SNPs. Also the GWAS quality has been monitored using hilum color and seed coat color. Fast genotyping method e.g., using a set of core SNP array is in high demand for genetic study or molecular breeding (Chaudhary et al., 2015).

The information gained in this study demonstrated that the usefulness of the medium-density SNP array in genotyping for genetic study and molecular breeding.

Up to date, there are a large number of loci or QTL have been identified by GWAS using different set of natural population or by linkage or association mapping using biparental populations under different environments in different years. In generally, the effect of each locus is rather small, its detection might be influenced by population size, population structure, accuracy of phenotyping, physical location of the causal gene (e.g., pericentromeric region), epistatic association between QTLs as well as environmental factors. High negative correlation coefficients between oil and protein content in soybean was revealed in this study, which is consistent with previous reports (Boydak et al., 2002; Karaaslan et al., 2008); common regions or loci might have favorable effect on one and unfavorable effect on the other. The higher negative correlation coefficients of two traits might reflect that we might be able to detect QTL or QTN with higher effect on both traits. Hwang et al. (2014) found seven of 13 regions associated with oil content also have effect on protein content (Hwang et al., 2014). Similarly, in this study, we have detected 11 common QTNs associated with oil and antagonistically associated with protein, although no universal QTN detected over all environments. However, the overall oil and protein content can be varied to a great extent, also the environmental effect e.g., latitudinal location, temperature can also influence the balance of two contents, there are a lot loci affecting most to one content, but not the other, at least not significantly (Eskandari et al., 2013).

Overall, a large number of loci have been identified to underlie some important agronomic traits e.g., flowering time, maturity, oil and protein contents; however, a detailed study may only detect some of them. Ideally, a large numbers of natural population can be subtracted into a subpopulation each member of which carries higher or lower phenotypic values for a given trait; GWAS for the given trait can be performed using in this subpopulation (Yan et al., 2017).

A large number of QTLs or loci underlying agronomically important traits have been identified by GWAS or linkage mapping, some of which were detected in different environments or in different populations while some are environmental or population specific. Although molecular identities of genes or QTL underlying some important agronomic traits e.g., maturity have been disclosed, vast of loci underlying quantitative traits like soybean seed protein /oil content are still largely unknown. GWAS in combination with biparental populations such as RIL, NIL, CSSL, is very powerful for QTL identification and their gene cloning. As high throughput sequencing data aggregate, the important QTL or QTN detected by traditional linkage mapping or GWAS will be verified and subsequently cloned. As most components of a molecular or signaling pathway have been identified (Gentzbittel et al., 2015), information of gene regulation or crosstalk with different pathways will enable us to build a genetic network that can be used in molecular design breeding.

## Author contributions

ZX conceived this project; YW, YL performed the most experiments in the laboratory; HW, BH, HZ, SL, XL, XC, HQ, JY, CZ, DH conducted field experiment and phenotypic observation; JZ, ZW, ZX: performed data analysis including GWAS; ZX, YW, and YL wrote the article; DW contributed to scientific discussions and critical revision of manuscript. All authors reviewed the final manuscript.

### Conflict of interest statement

The authors declare that the research was conducted in the absence of any commercial or financial relationships that could be construed as a potential conflict of interest.

## Supplementary material

Figure S1 The frequency of heterozygous and linkage disequilibrium decade were culculated using Gapit. **(a)** The frequency of heterozygous nature was calculated for both individuals and markers. High level of heterozygosis indicated low quality. **(b)** Linkage disequilibrium are measured as R square for pair wise markers and plotted against their distance.

Figure S2 Phenotypic variations in maturity (R7, Beginning Maturity, R8, Full Maturity) of cultivars or accessions at different locations and in 2011 and 2012. The phenotypic segregation is shown in box-plot format. The interquartile region, median, and range are indicated by the box, the bold horizontal line, and the vertical line, respectively. For location, HRB, Harbin; MDJ, Mudanjiang; GZL, Gongzhuling; JN, Jinan; HA, Huaian; NJ, Nanjing. For years, 11, 2011; 12, 2012.

Figure S3 GWAS of flowering time (R1) in the northern geographic region using FarmCPU. Manhattan plots (bottom) and Quantile-quantile (upper right) plot for a trait. Negative log10 P-values from a genome-wide scan are plotted against SNP positions of 20 chromosomes. The horizontal dash line indicates the significant threshold (2 × 10^−5^). **(a)** Gongzhuling in 2011; **(b)** Gongzhuling in 2012; **(c)** Harbin in 2011; **(d)** Harbin in 2012; **(e)** Mudanjiang in 2011; **(F)** Mudanjiang in 2012.

Figure S4 GWAS of flowering time (R1) in the southern geographic region using FarmCPU. Manhattan plots (bottom) and Quantile-quantile (upper right) plot for a trait. Negative log10 *P*-values from a genome-wide scan are plotted against SNP positions of 20 chromosomes. The horizontal dash line indicates the significant threshold (2 × 10^−5^). **(a)** Jinan in 2011; **(b)** Jinan in 2012; **(c)** Huaian in 2011; **(d)** Huaian in 2012; **(e)** Nanjing in 2011; **(f)** Nanjing in 2012.

Figure S5 GWAS of beginning maturity, R7, flowering time (R1) using FarmCPU. Manhattan plots (left) and Quantile-quantile (right) plot. Negative log10 *P*-values from a genome-wide scan are plotted against SNP positions of 20 chromosomes. The horizontal dash line indicates the significant threshold (2 × 10^−5^). **(a)** Gongzhuling in 2011; **(b)** Gongzhuling in 2012; **(c)** Mudanjiang 2011; **(d)** Mudanjiang in 2012; **(e)** Jinan in 2011; **(f)** Huaian in 2011; **(g)** Huaian in 2012; **(h)** Nanjing in 2011; **(i)** Nanjing in 2012.

Figure S6 GWAS of full maturity, R8, using FarmCPU. Manhattan plots (left) and Quantile-quantile (right) plot. Negative log10 *P*-values from a genome-wide scan are plotted against SNP positions of 20 chromosomes. The horizontal dash line indicates the significant threshold (2 × 10^−5^). **(a)** Gongzhuling in 2011; **(b)** Gongzhuling in 2012; **(c)** Mudanjiang 2011; **(d)** Mudanjiang in 2012; **(e)** Jinan in 2011; **(f)** Huaian in 2011.

Figure S7 GWAS of oil contents using FarmCPU. Manhattan plots (left) and Quantile-quantile (right) plot. Negative log10 *P*-values from a genome-wide scan are plotted against SNP positions of 20 chromosomes. The horizontal dash line indicates the significant threshold (2 × 10^−5^). **(a)** Harbin in 2011; **(b)** Harbin in 2012; **(c)** Mudanjiang 2011; **(d)** Mudanjiang in 2012; **(e)** Jinan in 2011; **(F)** Jinan in 2012; **(g)** Huaian in 2011; **(h)** Huaian in 2012; **(i)** Nanjing in 2011.

Figure S8 GWAS of protein contents using FarmCPU. Manhattan plots (left) and Quantile-quantile (right) plot. Negative log10 *P*-values from a genome-wide scan are plotted against SNP positions of 20 chromosomes. The horizontal dash line indicates the significant threshold (2 × 10^−5^). **(a)** Harbin in 2011; **(b)** Harbin in 2012; **(c)** Mudanjiang 2011; **(d)** Mudanjiang in 2012; **(e)** Jinan in 2011; **(F)** Jinan in 2012; **(g)** Huaian in 2011; **(h)** Huaian in 2012; **(i)** Nanjing in 2011.

Table S1 Geographic origins of soybean cultivars or accession used in this study.

Table S2 The correlation coefficients between R1 (first flower), R7 (beginning of maturity) and R8 (Fully Maturity) of soybean cultivars grown at different locations in 2011 or 2012.

Table S3 QTNs for flowering time (R1) and maturity (R7 or R8) were detected using FarmCPU.

Table S4 QTNs for protein and oil contents were detected using FarmCPU.

Data Sheet 1 Raw data and probe information of SoySNP8k iSelect BeadChip, which can be download at.ftp://159.226.208.134/public/SNP_data.zip.
