# Supplementary figures and images for: Genotyping of Soybean Cultivars With Medium-Density Array Reveals the Population Structure and QTNs Underlying Maturity and Seed Traits
Source: Front Plant Sci. 2018 May 9;9:610. doi: 10.3389/fpls.2018.00610 (PMC5954420; doi:10.3389/fpls.2018.00610)

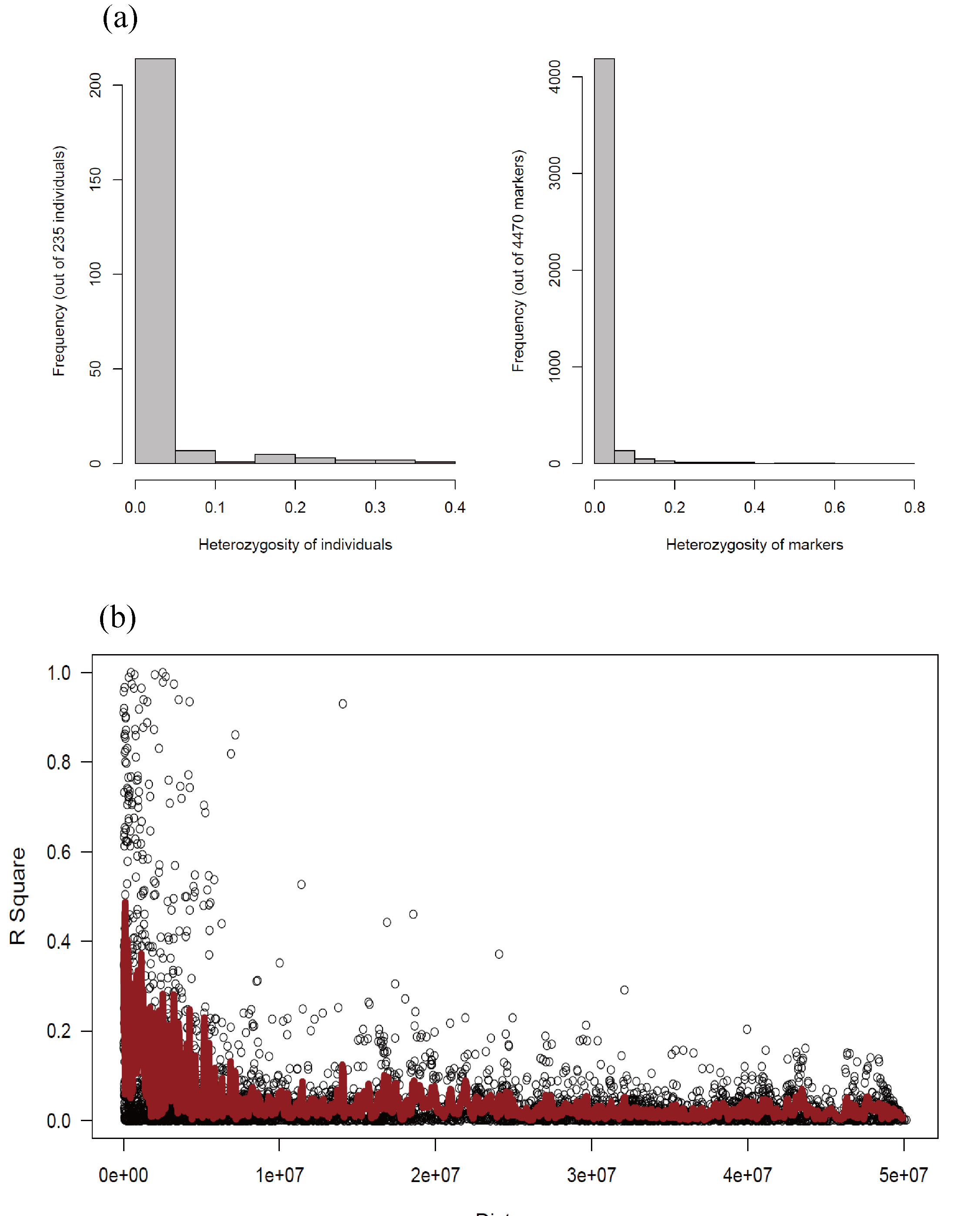

Supplement: Figure S1 — The frequency of heterozygous and linkage disequilibrium decade were culculated using Gapit. (a) The frequency of heterozygous nature was calculated for both individuals and markers. High level of heterozygosis indicated low quality. (b) Linkage disequilibrium are measured as R square for pair wise markers and plotted against their distance. [file Image_1.jpg]

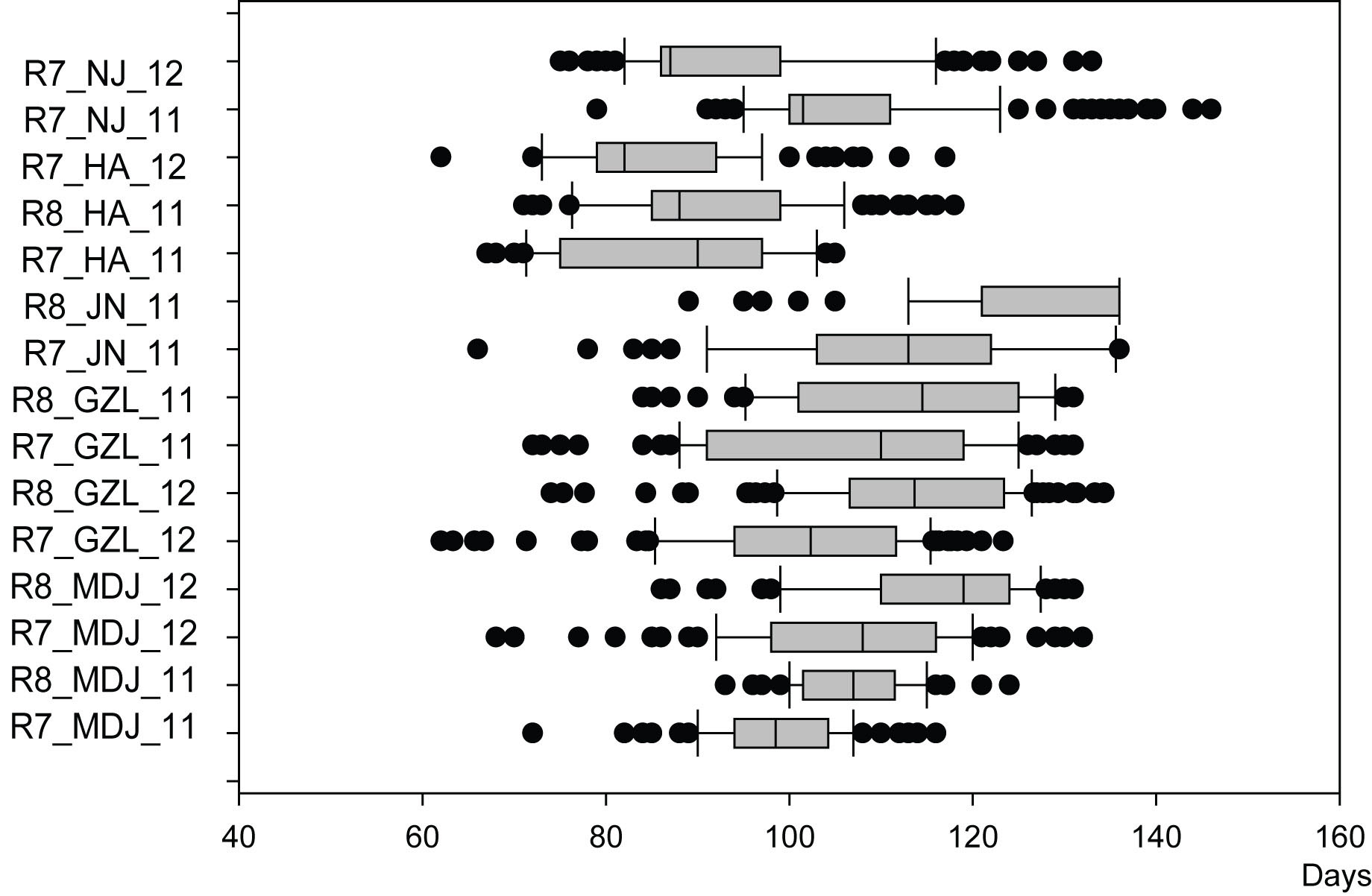

Supplement: Figure S2 — Phenotypic variations in maturity (R7, Beginning Maturity, R8, Full Maturity) of cultivars or accessions at different locations and in 2011 and 2012. The phenotypic segregation is shown in box-plot format. The interquartile region, median, and range are indicated by the box, the bold horizontal line, and the vertical line, respectively. For location, HRB, Harbin; MDJ, Mudanjiang; GZL, Gongzhuling; JN, Jinan; HA, Huaian; NJ, Nanjing. For years, 11, 2011; 12, 2012. [file Image_2.jpg]

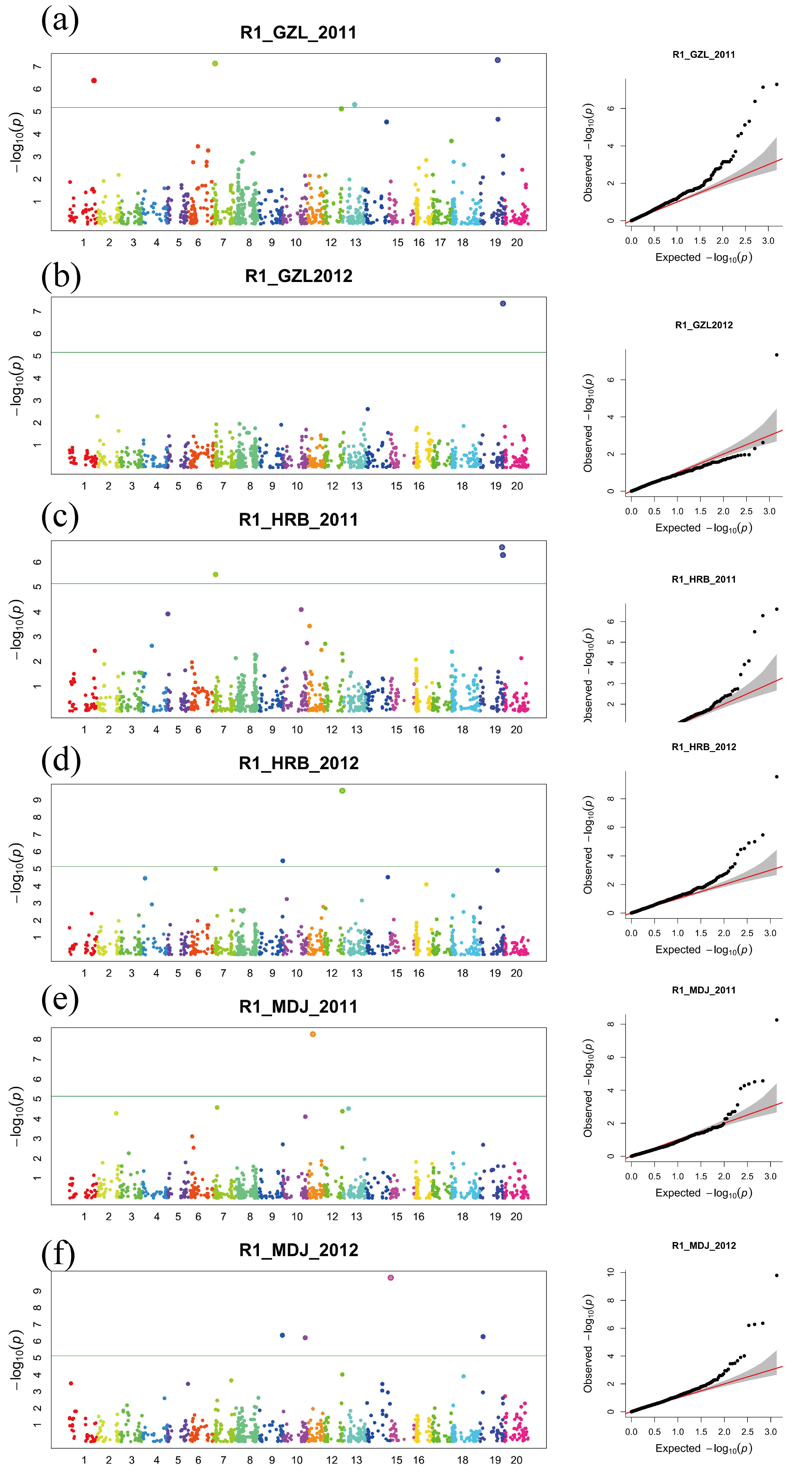

Supplement: Figure S3 — GWAS of flowering time (R1) in the northern geographic region using FarmCPU. Manhattan plots (bottom) and Quantile-quantile (upper right) plot for a trait. Negative log10 P-values from a genome-wide scan are plotted against SNP positions of 20 chromosomes. The horizontal dash line indicates the significant threshold (2 × 10^−5^). (a) Gongzhuling in 2011; (b) Gongzhuling in 2012; (c) Harbin in 2011; (d) Harbin in 2012; (e) Mudanjiang in 2011; (F) Mudanjiang in 2012. [file Image_3.JPEG]

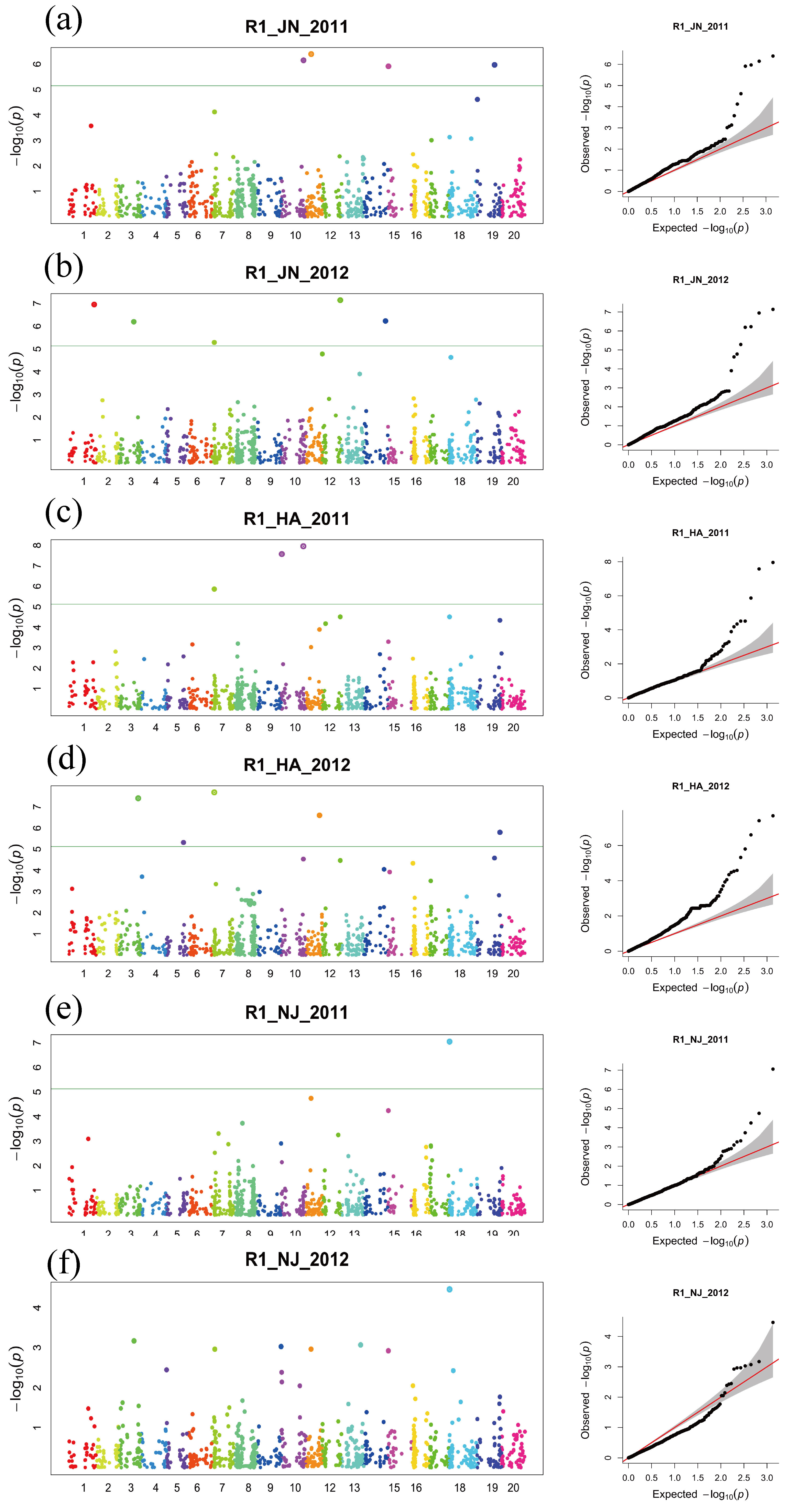

Supplement: Figure S4 — GWAS of flowering time (R1) in the southern geographic region using FarmCPU. Manhattan plots (bottom) and Quantile-quantile (upper right) plot for a trait. Negative log10 P-values from a genome-wide scan are plotted against SNP positions of 20 chromosomes. The horizontal dash line indicates the significant threshold (2 × 10^−5^). (a) Jinan in 2011; (b) Jinan in 2012; (c) Huaian in 2011; (d) Huaian in 2012; (e) Nanjing in 2011; (f) Nanjing in 2012. [file Image_4.JPEG]

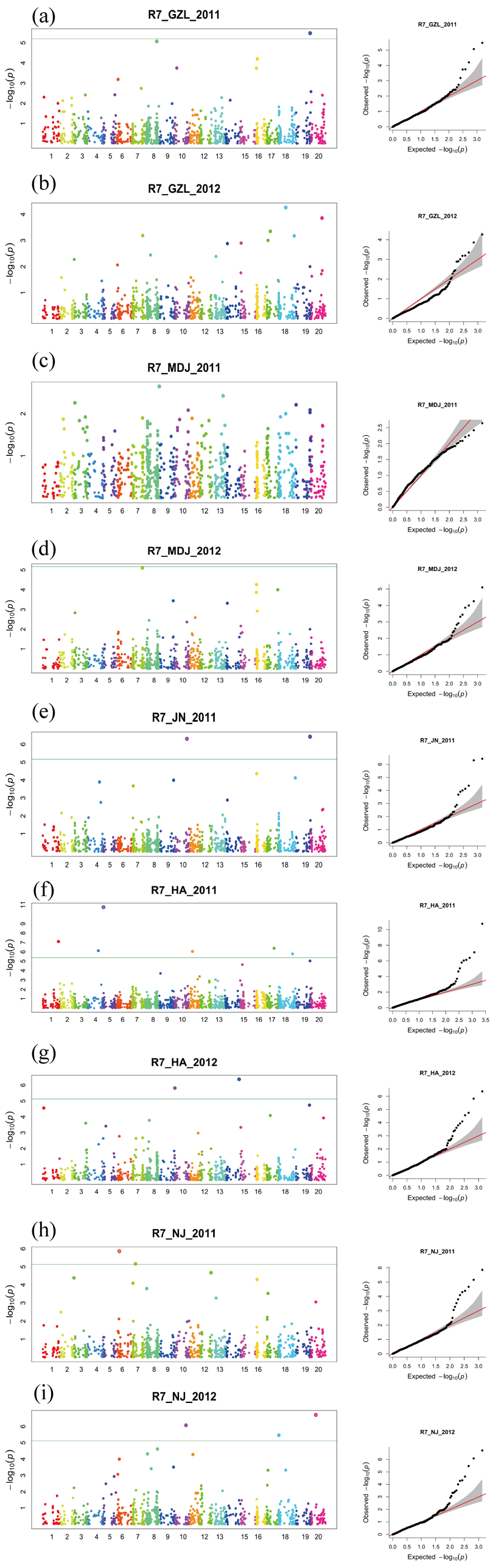

Supplement: Figure S5 — GWAS of beginning maturity, R7, flowering time (R1) using FarmCPU. Manhattan plots (left) and Quantile-quantile (right) plot. Negative log10 P-values from a genome-wide scan are plotted against SNP positions of 20 chromosomes. The horizontal dash line indicates the significant threshold (2 × 10^−5^). (a) Gongzhuling in 2011; (b) Gongzhuling in 2012; (c) Mudanjiang 2011; (d) Mudanjiang in 2012; (e) Jinan in 2011; (f) Huaian in 2011; (g) Huaian in 2012; (h) Nanjing in 2011; (i) Nanjing in 2012. [file Image_5.JPEG]

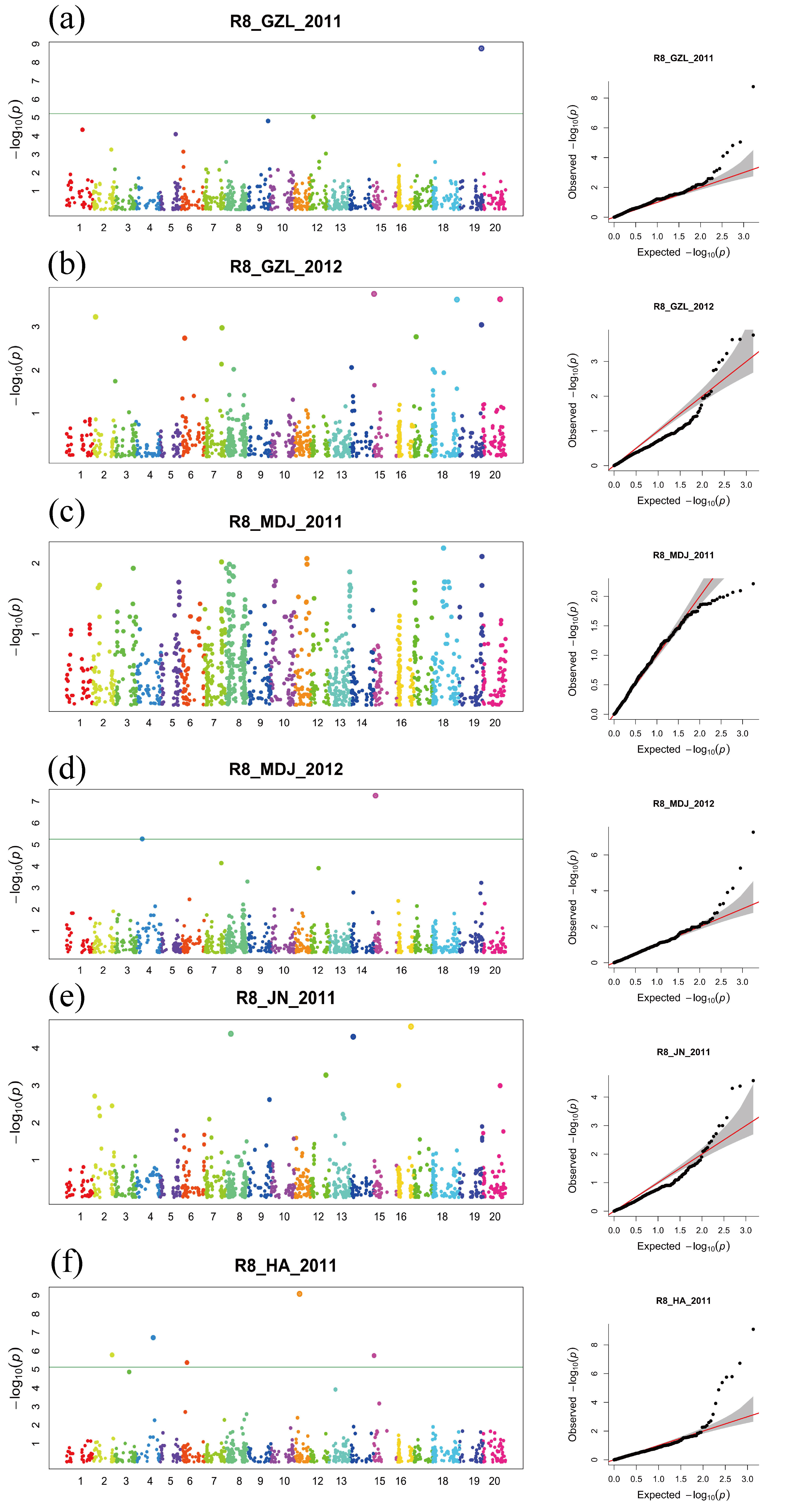

Supplement: Figure S6 — GWAS of full maturity, R8, using FarmCPU. Manhattan plots (left) and Quantile-quantile (right) plot. Negative log10 P-values from a genome-wide scan are plotted against SNP positions of 20 chromosomes. The horizontal dash line indicates the significant threshold (2 × 10^−5^). (a) Gongzhuling in 2011; (b) Gongzhuling in 2012; (c) Mudanjiang 2011; (d) Mudanjiang in 2012; (e) Jinan in 2011; (f) Huaian in 2011. [file Image_6.JPEG]

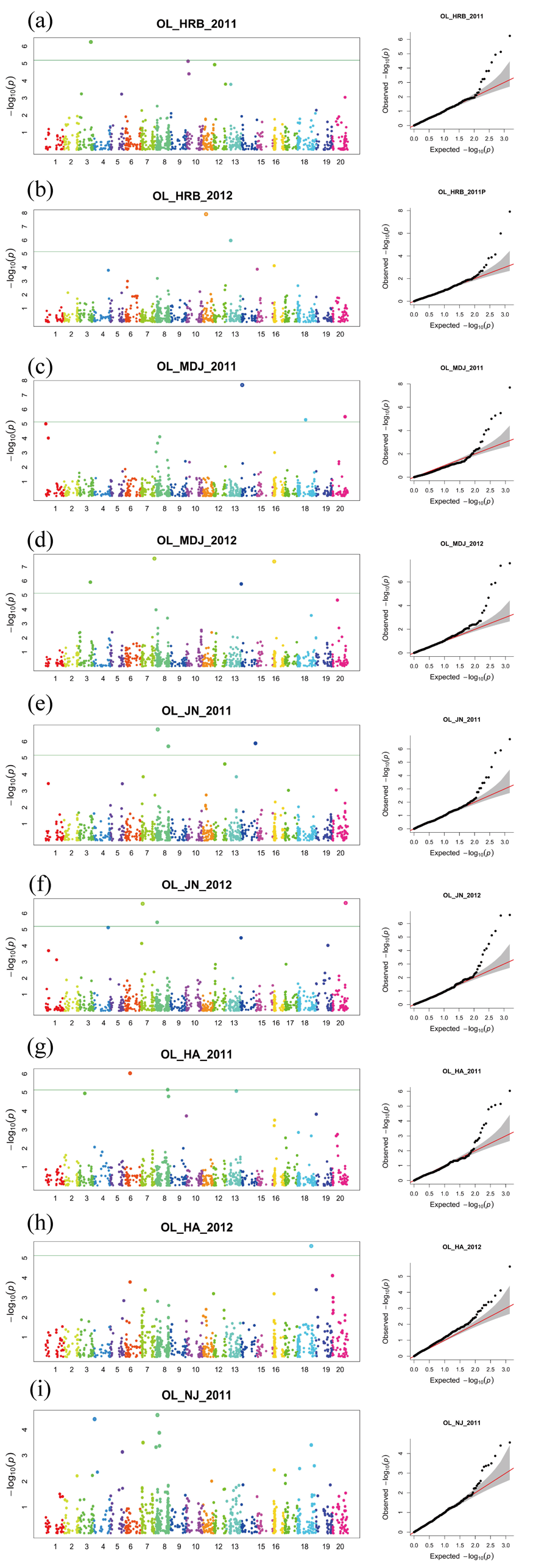

Supplement: Figure S7 — GWAS of oil contents using FarmCPU. Manhattan plots (left) and Quantile-quantile (right) plot. Negative log10 P-values from a genome-wide scan are plotted against SNP positions of 20 chromosomes. The horizontal dash line indicates the significant threshold (2 × 10^−5^). (a) Harbin in 2011; (b) Harbin in 2012; (c) Mudanjiang 2011; (d) Mudanjiang in 2012; (e) Jinan in 2011; (F) Jinan in 2012; (g) Huaian in 2011; (h) Huaian in 2012; (i) Nanjing in 2011. [file Image_7.JPEG]

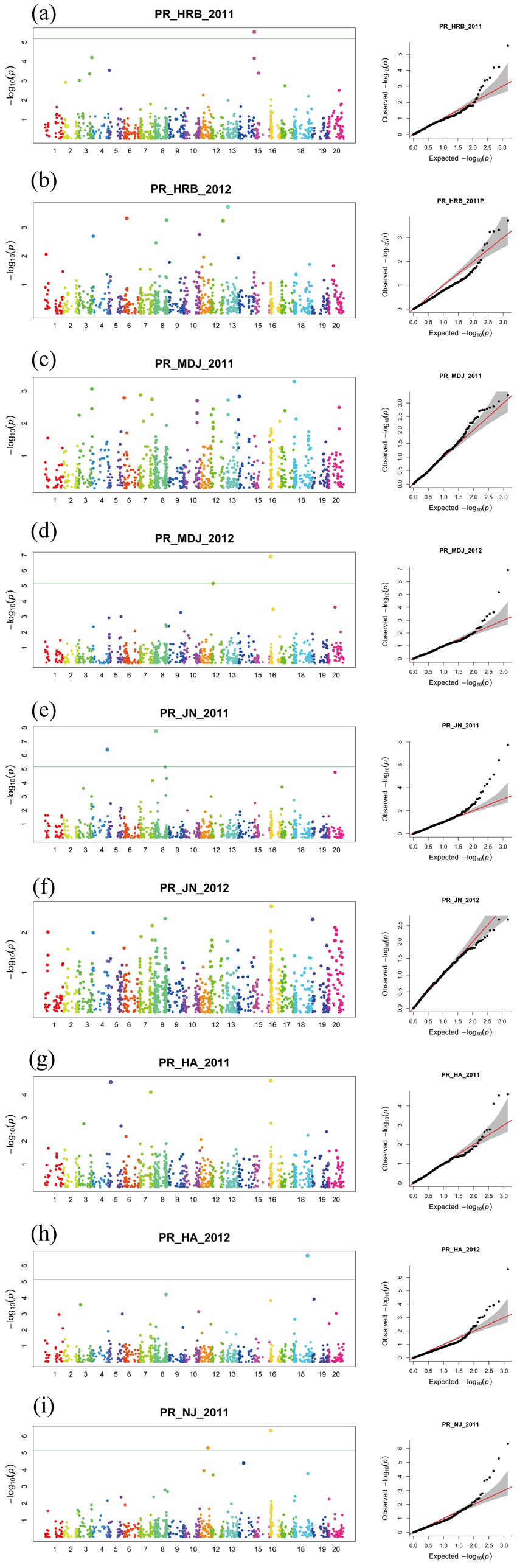

Supplement: Figure S8 — GWAS of protein contents using FarmCPU. Manhattan plots (left) and Quantile-quantile (right) plot. Negative log10 P-values from a genome-wide scan are plotted against SNP positions of 20 chromosomes. The horizontal dash line indicates the significant threshold (2 × 10^−5^). (a) Harbin in 2011; (b) Harbin in 2012; (c) Mudanjiang 2011; (d) Mudanjiang in 2012; (e) Jinan in 2011; (F) Jinan in 2012; (g) Huaian in 2011; (h) Huaian in 2012; (i) Nanjing in 2011. [file Image_8.JPEG]
