# Supplementary material for: Genotyping of Soybean Cultivars With Medium-Density Array Reveals the Population Structure and QTNs Underlying Maturity and Seed Traits
Source: Front Plant Sci. 2018 May 9;9:610. doi: 10.3389/fpls.2018.00610 (PMC5954420; doi:10.3389/fpls.2018.00610)
Supplement: Data Sheet 1 — Raw data and probe information of SoySNP8k iSelect BeadChip, which can be download at.ftp://159.226.208.134/public/SNP_data.zip. [file Data_Sheet_1.ZIP › Description for Zip files.docx]

**Supplementary Material: SNP_data.zip**

**Genotyping of soybean cultivars with medium-density array reveals the population structure and QTNs underlying maturity and seed traits**

**WANG** **Ya-ying**^1,2,^ **^ψ^**, **LI Yu-qiu**^1,2,5,^ **^ψ^**, **WU Hong-yan**^1^, **HU Bo**^1,2^, **ZHENG Jia-jia**^1,2^, **ZHAI Hong**^1^, **L**v **Shi-xiang**^1,3^, **Liu Xin-lei**^3^, **Chen Xin**^4^, **Qiu Hong-me**i^5^, **Yang Jiayin**^6^, **Zong Chun-mei**^7^, **Han De-zhi**^8^, **WEN Zi-xiang**^9^, **WANG De-chun**^9^, **XIA Zheng-jun**^*1^,

**Enlosed:**

**File 1:**

**File name: Probe_information**

**Description:** **Probe information of SoySNP8k iSelect BeadChip**

**File 2:**

**File name:** SNP_raw_data.txt

**Description:** SNP raw data generated for for 235 cultivar with all 7189 SNP postions.

**File 3:** 4471_Polymorphic_Clean_data.txt

Description: This data was generated from File 1 (SNP_raw_data.txt) by removing non polymorphic and batch specific SNP.

**File 4:**

**File name:** 4471_Polymorphic_SNP_Position

Description: The genomic postions in reference Williams 82(Gmax_275_Wm82.a2.v1, <http://phytozome.jgi.doe.gov/pz/portal.html#!info?alias=Org_Gmax>) for 4471 polymorphic SNP in file 2 (4471_Polymorphic_Clean_data.txt).
